# Hierarchical Flux‐Protection Engineering Stabilizes Oxidation‐Prone Metabolic Nodes for High‐Level Curcumin Biosynthesis

**DOI:** 10.1002/advs.77628

**Published:** 2026-09-13

**Authors:** Jianbin Chen, Qihang Chen, Shurong Zhang, Yajuan Su, Xiaoyi Zou, Ke Wang, Shike Liu, Qian He, Liang Zhang, Weizhu Zeng, Jingwen Zhou

**Affiliations:** ^1^ Engineering Research Center of Ministry of Education on Food Synthetic Biotechnology Jiangnan University Wuxi Jiangsu China; ^2^ Science Center for Future Foods Jiangnan University Wuxi Jiangsu China; ^3^ Jiangsu Province Engineering Research Center of Food Synthetic Biotechnology Jiangnan University Wuxi China

**Keywords:** carbon flux efficiency, curcumin biosynthesis, flux redistribution, oxidation‐prone intermediate, polyphenolic natural product, redox‐sensitive intermediate, respiratory chain modulation

## Abstract

Biosynthesis of complex natural products in engineered microbial hosts is frequently constrained by unstable oxidation‐prone intermediates that disrupt metabolic balance and lower carbon flux efficiency. In the synthesis pathways of polyphenolic compounds, redox‐sensitive nodes often function as bottlenecks due to intermediate accumulation and spontaneous oxidation under aerobic conditions. In this study, the oxygen‐dependent degradation of L‐3,4‐dihydroxyphenylalanine (L‐DOPA), a redox‐sensitive intermediate in curcumin biosynthesis, was characterized, and a hierarchical strategy was implemented to stabilize oxidation‐prone nodes while rebalancing intracellular carbon and electron fluxes. Adaptive evolution improved host robustness, respiratory chain modulation redistributed reducing equivalents under oxygen limitation, and directed evolution of 4‐hydroxyphenylacetate 3‐monooxygenase (HpaB) rebalanced intermediate distribution and enhanced productive hydroxylation. Combined with multistage pH–DO control, these strategies markedly improved carbon utilization efficiency and curcumin biosynthesis. In 5 L fed‐batch fermentation, curcumin production reached 1075.2 mg/L, the highest reported titer for single‐strain *de novo* curcumin biosynthesis. Extension of this framework to caffeic acid production increased the titer by approximately 69.1% and reduced A_400_‐associated browning, supporting transferability to another oxidation‐prone phenolic product. This study demonstrates the effectiveness of flux protection in curcumin biosynthesis and supports the potential transferability of this framework to related HpaB‐dependent phenylpropanoid pathways.

## Graphical abstract

Flux‐protection engineering stabilizes an oxidation‐prone L‐DOPA node in engineered *E. coli* by combining acid‐adapted chassis evolution, respiratory‐chain modulation, enzyme‐level flux matching, and pH–DO control. Carbon is redirected from oxidative browning to productive biosynthesis, enabling 1075.2 mg/L curcumin and demonstrating transferability to caffeic acid production.

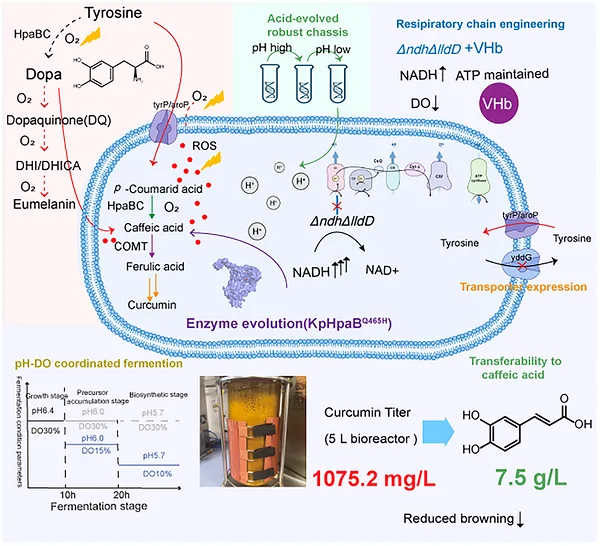

## 1 Introduction

Oxidation‐prone metabolic nodes and their unstable intermediates constitute a persistent bottleneck in microbial biosynthesis of polyphenolic and other redox‐active natural products [1]. During aerobic fermentation, many high‐value pathways generate catechol‐containing or otherwise oxidation‐sensitive intermediates susceptible to spontaneous oxidation, leading to structural degradation, pigment formation, reactive oxygen species accumulation, and irreversible carbon loss [2]. Oxidation‐associated instability decreases product titer and process robustness [3], complicates downstream purification, and increases manufacturing costs [4], thereby weakening the industrial competitiveness of biobased production routes [5]. In some cases, oxidation‐induced browning and by‐product formation can directly impair product quality and limit scale‐up feasibility [6, 7]. Therefore, beyond simply reinforcing pathway flux, stabilizing vulnerable metabolic nodes and minimizing oxidation‐driven flux dissipation are increasingly important design principles for robust microbial cell factories [8, 9]. This issue is particularly critical for polyphenolic products, whose biosynthetic performance is often constrained not only by enzyme activity and precursor availability, but also by the intrinsic chemical instability of pathway intermediates [10].

During phenolic acid biosynthesis, catechol‐containing intermediates such as L‐3,4‐dihydroxyphenylalanine (L‐DOPA) are highly susceptible to oxidative degradation, and their accumulation often causes substantial carbon loss [11]. Similarly, caffeic acid is also prone to oxidative browning during biosynthesis, limiting its production efficiency [12]. This problem is also highly relevant to curcumin biosynthesis; oxidation‐associated browning of the fermentation broth indicates that pathway intermediates may undergo spontaneous oxidation under production conditions [13]. However, efforts in microbial curcumin production have mainly focused on improving precursor availability [14], increasing malonyl‐CoA supply [15, 16], and optimizing pathway expression [17]. Additionally, coculture‐based strategies in *Escherichia coli*, incorporating molecular chaperone‐assisted protein folding, outer membrane vesicle‐mediated secretion, and optimized carbon source allocation have achieved curcumin titers up to 978 mg/L [18]. While these approaches effectively reinforce carbon flux toward curcumin formation, they do not directly address the instability of oxidation‐prone intermediates. As a result, oxidation‐driven carbon dissipation can prevent achieving both high titer and high robustness in de *novo* curcumin biosynthesis.

Several approaches have been explored to alleviate oxidation‐associated intermediate loss, all with notable limitations [2]. Direct strategies, such as supplementation with antioxidants or reducing agents such as butylated hydroxytoluene (BHT), can suppress oxidative degradation to some extent, but they do not resolve intracellular carbon leakage or redox imbalance and may obscure the contribution of engineered cellular mechanisms [19, 20]. Lowering dissolved oxygen (DO) levels can mitigate oxidation [21], but this often compromises respiratory metabolism and intracellular reducing power supply, which is generally unfavorable for target‐directed biosynthesis [22]. Persistent foaming in the 5 L bioreactor impaired oxygen transfer, while acidification during fermentation generated a coupled low‐pH and oxygen‐limited production environment [22, 23, 24]. Indirect stabilization strategies have also been proposed, including pH adjustment to improve the stability of sensitive intermediates, but under such conditions, poor acid tolerance of engineered strains frequently becomes a new production constraint [25]. These observations indicate that single interventions rarely reconcile pathway efficiency, redox homeostasis, host robustness, and intermediate stability simultaneously. Therefore, a more hierarchical engineering framework is required to address oxidation‐prone bottlenecks from the multiple dimensions of pathway stability, host physiology, and redox balance.

In this study, a hierarchical flux‐protection framework was established to prevent oxidative loss of productive carbon flux at vulnerable metabolic nodes in engineered *E. coli* for curcumin biosynthesis. Unlike conventional flux amplification, which primarily aims to increase pathway throughput, flux protection emphasizes preserving carbon already directed into the productive pathway by stabilizing oxidation‐prone intermediates and reducing oxidation‐associated carbon loss. Accordingly, L‐DOPA was defined as an oxidation‐prone carbon‐leakage node through mechanistic analysis of its oxygen‐dependent degradation and A_400_‐associated browning. The pathway was then systematically reconstructed by combining pathway architectural redistribution, acid‐adapted chassis engineering, respiratory‐chain modulation, enzyme‐level flux matching, and multistage pH–DO control. This integrated strategy enabled high‐level curcumin production in a single‐strain *de novo* biosynthetic system and was further extended to caffeic acid production, supporting its transferability to another oxidation‐prone phenolic product.

## 2 Materials and Methods

### 2.1 Strains, Growth Media, and Chemicals

The JM109 *E. coli* strain was utilized for plasmid assembly, while *E. coli* BL21(DE3) was employed as the production host for curcumin biosynthesis. All engineered strains generated in this work are summarized in Table S1. Luria‐Bertani (LB) medium, consisting of 10 g/L NaCl, 5 g/L yeast extract, and 10 g/L tryptone, was used for strain activation, plasmid amplification, and seed culture preparation. The production medium used for shake‐flask cultivation and 5 L bioreactor fermentation was prepared according to a previously reported formulation [11]. For shake‐flask experiments, the medium was supplemented with 12 g/L CaCO_3_ as a buffering agent. Glucose was sterilized separately prior to addition, and an antibiotic cocktail (0.1%, v/v) including chloramphenicol (34 mg/mL), ampicillin (100 mg/mL), streptomycin (50 mg/mL), and kanamycin (50 mg/mL) was introduced when required. Standards of ferulic acid, curcumin, demethoxycurcumin, and dimethoxycurcumin were obtained from Weikeqi (Sichuan, China). Plasmid miniprep kits, DNA fragment purification kits, and primers were purchased from Sangon Biotech (Shanghai, China).

### 2.2 Plasmid Construction

The engineered strains generated in this work are summarized in Table S1. All overexpressed endogenous genes were amplified from *E. coli* BL21(DE3). Plasmid construction is illustrated using pACYCDuet‐T7‐*FjTAL*‐*KpHpaBC*‐T7‐*AtCOMT1* as an example. The *HpaB* and associated coupling protein (*HpaC*) gene fragment from *Klebsiella planticola* (GenBank: CP180442.1; WP_277137326.1) was amplified from a synthesized DNA template using primers CDF‐M2‐F and CDF‐M2‐R. The previously constructed plasmid pACYCDuet‐T7‐*FjTAL*‐*EcHpaBC*‐T7‐*AtCOMT1* [13] was linearized with primers pla‐F and pla‐R. Purified fragments of *KphpaBC* were attached to multiple cloning site 1 of linearized T7‐*AtCOMT‐*pACYCDuet‐T7‐*FjTAL*‐ using a Gibson Assembly Kit (New England Biolabs, Ipswich, MA, USA).

### 2.3 Genome Modification

Deletion of the pyruvate kinase 1 (pykF) gene was carried out using a CRISPR‐Cas9 dual‐plasmid genome editing system as previously described [26]. To knockout *pykF*, the pCas9 plasmid was transformed into host cells. An online tool (https://benchling.com) was then used to predict the single guide RNA (sgRNA) of the target gene, and a pTarget carrying N20 was constructed. Upstream homologous fragments of the target gene were amplified with primers pykF‐UP‐F and pykF‐UP‐R. Downstream homologous fragments were amplified with primers pykF‐DOWN‐F and pykF‐DOWN‐R. Fusion fragments were obtained with primers pykF‐UP‐F and pykF‐DOWN‐R. Finally, both pTarget and homologous fragments were electroporated into competent cells carrying pCas9, spread on LB agar containing kanamycin and streptomycin, and incubated for 16 h at 37°C. Single colonies were verified with primers pykF‐DOWN‐YZ‐F and pykF‐DOWN‐YZ‐R. In this study, the same method was used to knock out other genes. For chromosomal integration of phosphoenolpyruvate (PEP) synthase gene *ppsA* at *pykF*, the homologous arms were replaced by those flanking the *pykF* site, and a T7 promoter‐driven *ppsA* expression cassette was inserted between the modified homologous fragments. Subsequent experimental procedures were identical to those described above. The caffeic acid‐producing strains CA24–CA30 were constructed as a stepwise matched‐strain series from a common parental background. Adjacent strains retained the same precursor‐supply and caffeic acid pathway‐expression modules and differed only in the engineering layer indicated in Table 1 and Table S1.

**TABLE 1:** Sequential comparison of product formation and A_400_‐based oxidation‐associated browning across engineered strains and process conditions.

**Scale**	**Strain**	**Key strategy**	**Product**	**Titer (mg/L)**	**A_400_**	**FPI**	**rFPI_i_**
Shake flask	Cur59	WT	Curcumin	55.87	1.74	32.05 ± 2.60	1.00
Shake flask	Cur84	ALE	Curcumin	93.31	1.79	52.22 ± 0.74	1.63
Shake flask	Cur95	*Kp*HpaBC (Enzyme‐level flux balancing)	Curcumin	111.34	2.16	51.55 ± 1.51	0.99
Shake flask	Cur118	Δ*ndh*/Δ*lldD* (Respiratory chain regulation)	Curcumin	155.27	1.89	82.15 ± 3.20	1.59
Shake flask	Cur123	VHb (Oxygen transfer control)	Curcumin	153.84	1.72	89.29 ± 3.37	1.09
Shake flask	Cur126	Transporter (Transport optimization)	Curcumin	165.55	1.80	91.97 ± 2.48	1.03
Shake flask	Cur132	Q465H (Enzyme‐level flux balancing)	Curcumin	233.76	1.13	206.87 ± 6.95	2.25
5 L bioreactor	Cur84	Original process	Curcumin	523.56	5.10	102.76 ± 3.82	1.00
5 L bioreactor	Cur132	Original process	Curcumin	762.69	3.28	232.81 ± 14.76	2.27
5 L bioreactor	Cur132	pH–DO	Curcumin	1075.2	0.81	1320.88 ± 19.76	5.67
Shake flask	CA24	WT	Caffeic acid	382.41	7.39	51.73 ± 5.45	1.00
Shake flask	CA25	ALE	Caffeic acid	442.60	7.01	63.13 ± 2.97	1.22
Shake flask	CA26	*Kp*HpaBC (Enzyme‐level flux balancing)	Caffeic acid	502.58	7.27	69.10 ± 1.46	1.09
Shake flask	CA27	Δ*ndh*/Δ*lldD* (Respiratory chain regulation)	Caffeic acid	698.59	6.17	113.28 ± 6.56	1.64
Shake flask	CA28	Q465H (Enzyme‐level flux balancing)	Caffeic acid	775.95	5.30	146.35 ± 1.06	1.29
Shake flask	CA29	VHb (Oxygen transfer control)	Caffeic acid	795.39	5.108	155.87 ± 3.65	1.06
Shake flask	CA30	Transporter (Transport optimization)	Caffeic acid	850.40	4.62	184.11 ± 8.72	1.18
5 L bioreactor	CA24	Original process	Caffeic acid	4435.49	17.83	248.81 ± 24.23	1.00
5 L bioreactor	CA30	Original process	Caffeic acid	6235.19	15.00	415.73 ± 29.51	1.67
5 L bioreactor	CA30	pH–DO	Caffeic acid	7499.62	11.97	626.64 ± 5.35	1.51

### 2.4 Culture Conditions

Shake‐flask fermentation was performed as described in our previous study [13]. Bench‐scale fed‐batch fermentation was conducted in a 5 L bioreactor with a working volume of 2.5 L. The seed culture was prepared as described above and inoculated at 8% (v/v). During fermentation, the temperature was maintained at 37°C, the airflow was set at 2 L/min, and DO was initially controlled at 30% with agitation speed ranging from 300 to 900 rpm. When the optical density at 600 nm (OD_600_) reached 30, the temperature was reduced to 30°C and 0.1 mm Isopropyl‐beta‐D‐thiogalactopyranoside (IPTG) was added to induce gene expression. Based on previously established fermentation strategy and the physiological characteristics of different production phases, a physiology‐guided stage‐wise pH–DO control strategy was developed [13]. During the initial growth phase, pH 6.4 and DO 30% were maintained to support biomass accumulation. Upon induction at OD_600_ = 30, the conditions were shifted to pH 6.0 and DO 15%. At 20 h, the pH and DO were further decreased to 5.7 and 10%, respectively, for the subsequent production phase. When DO began increasing, the feeding medium was supplied at a constant rate of 20 mL/h [13]. To evaluate the transferability of the flux‐protection framework, the integrated strain‐ and process‐engineering strategy was applied to caffeic acid production in a 5 L bioreactor. The fermentation process was performed based on a previously reported strategy [12]. Caffeic acid concentration, cell growth, and A_400_‐associated browning burden were monitored during fermentation. For no‐ascorbic‐acid controls, ascorbic acid was omitted from both the production medium and the fed‐batch feeding medium, while all other cultivation parameters were kept unchanged.

### 2.5 Analysis of L‐Dopa Oxidative Degradation Products

L‐DOPA oxidative degradation products were analyzed using an Orbitrap Eclipse Tribrid high‐resolution mass spectrometer. Briefly, L‐DOPA‐supplemented culture samples were collected at the indicated time points and centrifuged. The supernatant was diluted with an equal volume of acetonitrile, whereas the cell pellet was extracted with methanol/1% formic acid in water (7:3, v/v) on ice for 30 min. After centrifugation, the two supernatant fractions were combined, filtered through a 0.22 µm membrane, and subjected to MS analysis. Metabolites were detected in both positive and negative ion modes using data‐dependent acquisition (DDA). Raw data were processed using Compound Discoverer (CD, Thermo Fisher Scientific) for metabolite annotation and relative quantification.

### 2.6 Determination of A_400_‐Based Browning Burden

Oxidation‐associated browning was evaluated by measuring the absorbance of cell‐free fermentation supernatants at 400 nm (A_400_). Fresh medium was used for background correction. Full UV–vis spectra of major pathway‐related compounds, medium controls, and oxidized/browned samples were additionally examined to assess their contributions to absorbance at 400 nm. Because A_400_ represents a composite visible‐absorbance signal rather than a compound‐specific measurement, it was operationally defined as an A400‐based oxidation‐associated browning index.

### 2.7 Correlation Analysis Between L‐DOPA Consumption and A_400_

To evaluate the relationship between L‐DOPA oxidation and browning, L‐DOPA‐supplemented samples were incubated under the indicated conditions and collected at different time points. Residual L‐DOPA was quantified by HPLC, and L‐DOPA consumption was calculated relative to the initial concentration. Linear regression analysis was then performed between L‐DOPA consumption and A_400_.

### 2.8 Intracellular NADH/NAD^+^ Ratio and ATP Measurement

Fermentation samples were rapidly chilled, harvested by centrifugation at 4°C, and normalized according to cell biomass. NADH and NAD^+^ were extracted separately and quantified using an enzymatic cycling‐based NAD^+^/NADH assay. ATP content was measured using a luciferase‐based ATP assay according to the manufacturer's instructions. NADH/(NADH + NAD^+^) ratios and ATP contents were calculated from three biological replicates.

### 2.9 Calculation of Flux‐Protection Index

The flux‐protection index (FPI) was calculated as:

$$\mathrm{FP}I_{i}=P_{i}/A_{400i}$$

where P_i_ represents the product titer of strain or condition i, and A400_i_ represents the corresponding oxidation‐associated browning burden. The relative FPI (rFPI_i_) was calculated as:

$$\mathrm{rFP}I_{i}=\;\mathrm{FP}I_{i}/\mathrm{FP}I_{i-1}$$

where FPI_i‐1_ represents the FPI of the preceding strain or process condition within the same comparison group. An rFPI_i_ value greater than 1 indicates improved product output per unit browning burden, whereas an rFPI_i_ value lower than 1 indicates reduced flux‐protection efficiency. FPI values were calculated independently for three biological replicates and are reported as mean values with 95% confidence intervals.

### 2.10 Enzyme Kinetic Assays of *Kp*HpaB Variants

Enzyme kinetic assays of KpHpaB variants, including *Kp*HpaB^WT^ and KpHpaB^Q465H^, were performed in a 500 µL reaction system containing Tris‐HCl buffer (pH 5.7, 6.0,6.4 and 7.5), substrate, NADH, purified *Kp*HpaB variant, and purified KpHpaC. The working concentrations of *Kp*HpaB variant and *Kp*HpaC were both 0.1 µm. Reactions were initiated by the addition of NADH and conducted under initial‐rate conditions. Samples were collected at predefined time points between 1 and 30 min and immediately quenched with ice‐cold methanol or 4 mm HCl, depending on the analyte. After centrifugation, the supernatants were filtered through a 0.22 µm membrane and analyzed by HPLC. Product formation was quantified using corresponding authentic standards. Initial reaction rates were calculated from the linear phase of product formation, and Km and Vmax were obtained by nonlinear fitting of the Michaelis–Menten equation. kcat was calculated based on the enzyme concentration used in the reaction, and catalytic efficiency was expressed as kcat/Km. All assays were performed in independent triplicates.

### 2.11 Atmospheric and Room Temperature Plasma (ARTP) Mutagenesis and Library Construction

Mutagenesis was performed using an ARTP mutation breeding system (Tianmu Biotechnology, Jiangsu, China) to construct an *E. coli* mutant library. Exponential phase cells were harvested, washed twice with sterile saline, and resuspended at a cell density of ∼ 10^7^–10^8^ colony‐forming units (CFU)/mL. Aliquots (10 µL) were spread onto sterile metal plates and exposed to helium plasma for varying durations (30–180 s). After treatment, cells were recovered in LB medium at 37°C for 1 h, followed by serial dilution and plating to determine CFU and calculate lethality rates. The lethality rate increased with exposure time and reached 97.6% at 60 s; therefore, considering the balance between mutation efficiency and cell viability, 60 s was selected as the optimal condition for subsequent mutant library construction and microbial microdroplet culture system (MMC)‐based high‐throughput screening.

### 2.12 Adaptive Acid Tolerance Evolution Using High‐Throughput MMC

Adaptive laboratory evolution (ALE) under acidic conditions was performed using high‐throughput MMC (Tianmu Biotechnology). The parental strain was grown overnight in LB medium, diluted to an initial OD_600_ of 0.05, and inoculated into fermentation medium before being loaded into the MMC device. Microdroplets (approximately 1–5 µL) were generated as independent microreactors to enable parallel growth screening.

Adaptive evolution was carried out through stepwise reduction of pH from 7.0 to 4.0. In each round, droplets were incubated at 37°C, and those exhibiting superior growth were automatically sorted by MMC and used to inoculate the next evolutionary cycle under lower pH conditions. After the final round, recovered cells were plated on LB agar plates, and individual colonies were evaluated in shake‐flask fermentation. Twelve evolved mutants with significantly improved growth under acidic conditions were obtained, and their growth, product biosynthesis, and organic acid accumulation were further evaluated under acidic fermentation conditions without carbonate buffering.

### 2.13 Integrated Multi‐Omics Analysis Under Acid Stress Conditions

Under acid stress conditions, engineered *E. coli* strains were cultivated in LB medium adjusted to pH 4.0 at 37°C with shaking at 220 rpm and harvested at the mid‐exponential phase (OD_600_ = 0.6–0.8). Fermentation samples were rapidly collected and centrifuged to remove the supernatant for metabolomic analysis. Cell pellets were washed three times with pre‐chilled phosphate‐buffered saline (PBS), immediately quenched in liquid nitrogen, and stored at −80°C. For transcriptomic analysis, RNA sequencing (RNA‐Seq) was performed by Genedenovo (Guangzhou, China). For proteomic analysis, cell pellets were lysed by ultrasonication on ice and centrifuged to remove cell debris. The supernatant was collected for protein quantification, followed by reduction with dithiothreitol (DTT), alkylation with iodoacetamide (IAA), and overnight digestion with trypsin. The resulting peptides were desalted using C18 extraction disks (CDS Analytical LLC, Oxford, PA, USA; Cat. No. 2215, 47 mm), lyophilized, and reconstituted prior to analysis. Both proteomic and metabolomic samples were analyzed using an Orbitrap mass spectrometry platform (Thermo Fisher Scientific, Waltham, MA, USA) in data‐independent acquisition (DIA) mode.

Raw transcriptomic data were processed using FASTP (v0.23.4) for quality control by removing adaptor sequences, low‐quality reads, and reads containing excessive ambiguous bases. Clean reads were aligned to rRNA sequences using Bowtie2 (v2.5.1) for rRNA removal, and remaining reads were mapped to the reference genome for gene expression quantification using d (v1.3.3). Sample reproducibility was evaluated by Pearson correlation analysis and principal component analysis (PCA). Differentially expressed genes were identified using edgeR (v3.42.4) with thresholds of |log_2_(fold change)| ≥ 1 and false discovery rate (FDR) < 0.05. Proteomic data were processed using the open‐source software DIA‐NN (v1.9.2) for spectral library searching and quantification, with FDR maintained below 1% at both precursor and protein levels. Integrated transcriptomic and proteomic analyses were conducted using Omicshare (Gene Denovo Biotechnology Co., Ltd., Guangzhou, China; https://www.omicshare.com/tools), including Gene Ontology (GO) and Kyoto Encyclopedia of Genes and Genomes (KEGG) enrichment analyses with FDR‐corrected *q*‐values < 0.05. Pearson correlation analysis was further performed to assess consistency between transcriptomic and proteomic datasets. In addition, gene set enrichment analysis (GSEA) was applied to identify significantly regulated functional pathways, providing a systems‐level understanding of acid tolerance mechanisms.

### 2.14 Molecular Docking and Molecular Dynamics Simulations

The three‐dimensional structures of Flavobacterium johnsoniae tyrosine ammonia lyase (*Fj*TAL) and *Klebsiella planticola* 4‐hydroxyphenylacetate 3‐monooxygenase (*Kp*HpaB) were predicted using AlphaFold3 (DeepMind, London, UK; https://alphafoldserver.com). The *Kp*HpaB^Q465H^ mutant model was reconstructed and optimized using Swiss‐Model (SIB Swiss Institute of Bioinformatics, Lausanne, Switzerland; https://swissmodel.expasy.org), with the wild‐type (WT) *Kp*HpaB structure as the template. Ligand structures, including L‐tyrosine, *p*‐coumaric acid, and flavin adenine dinucleotide (FADH), were constructed and energy‐minimized prior to molecular docking, which was performed using AutoDock Vina (v1.2.5). The docking grid was defined to cover the catalytic pocket and substrate access channel of *Fj*TAL or *Kp*HpaB. For each enzyme‐ligand complex, the docking pose with the lowest binding energy and a catalytically reasonable orientation was selected as the optimal docking result and used for subsequent structural analysis.

Molecular dynamics simulations were performed using GROMACS 2021.5. Each protein–ligand complex was placed in an explicit water box under periodic boundary conditions, and appropriate counterions were added to neutralize the system. The protonation state of H465 was evaluated using the APBS/PDB2PQR server and assigned as the doubly protonated histidine state (HSP), with both Nδ1 and Nε2 protonated. After energy minimization, the systems were equilibrated sequentially under NVT and NPT ensembles, followed by 500 ns production simulations. Long‐range electrostatic interactions were treated using the particle mesh Ewald method, and covalent bonds involving hydrogen atoms were constrained to allow a 2 fs integration time step. The resulting trajectories were analyzed for backbone root mean square deviation (RMSD), residue root mean square fluctuation (RMSF), hydrogen‐bond occupancy, substrate‐binding conformation, and Gibbs free‐energy landscapes. Representative stable conformations were extracted from trajectory frames corresponding to the global minimum‐energy basin of the Gibbs free‐energy landscape for structural comparison between KpHpaB^WT^ and KpHpaB^Q465H^.

### 2.15 Analytical Methods

All shake‐flask fermentation experiments were performed in independent triplicates, and fermentation broth samples were collected at 48 h unless otherwise stated. For fed‐batch fermentation, samples were collected at the indicated time point. Samples were appropriately diluted with methanol depending on the analytes, centrifuged, and supernatants were filtered through a 0.22 µm membrane prior to high‐performance liquid chromatography (HPLC) analysis. *p*‐coumaric acid, caffeic acid, and ferulic acid were detected at 323 nm, while curcumin was detected at 425 nm using an LC‐20A HPLC system (Shimadzu, Tokyo, Japan) equipped with a C18 column. The analytical procedures for these compounds and glycerol were consistent with those reported previously, with glycerol quantified using an Aminex HPX‐87H column and 5 mm H_2_SO_4_ as the mobile phase [13]. L‐tyrosine (L‐Tyr) and L‐DOPA were quantified using the same HPLC system equipped with a C18 column (4.6 mm × 250 mm). Fermentation samples were diluted with 3 m HCl to ensure concentrations within the standard curve range (0.1–1 g/L), followed by centrifugation (12 000 rpm, 5 min) and filtration (0.22 µm). The mobile phases consisted of water containing 0.1% trifluoroacetic acid (A) and methanol (B) at a flow rate of 1 mL/min, with a gradient program of 5% B from 0 to 2 min, 5%–25% B from 2 to 12 min, 25%–50% B from 12 to 17 min, and re‐equilibration to 5% B from 17 to 22 min. The column temperature was maintained at 30°C and detection was performed at 270 nm.

## 3 Results

### 3.1 Pathway Reconstruction Reveals L‐DOPA‐Mediated Oxidative Carbon Leakage in Curcumin Biosynthesis

**FIGURE 1:**
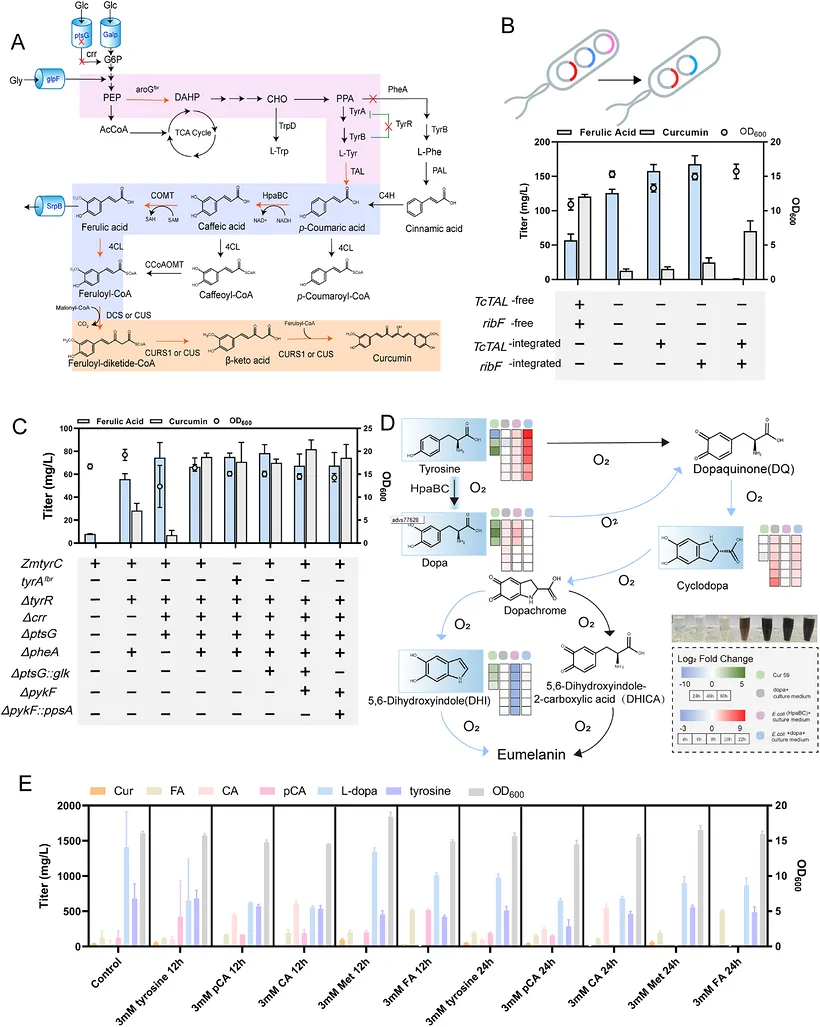
Metabolic flux redistribution and pathway optimization for *de novo* curcumin biosynthesis. (A) Engineered metabolic pathway for *de novo* curcumin biosynthesis in *E. coli*, including precursor supply optimization, phenolic acid biosynthesis, and curcumin formation modules. Key genetic modifications and pathway enzymes are indicated. (B) Effect of relocating non‐essential pathway genes (*TcTAL* and *ribF*) from plasmids to the chromosome using a high‐copy and integration dual‐track expression (HIDTE) strategy, in which essential high‐flux pathway modules are maintained on high‐copy plasmids, while auxiliary or non‐essential modules are chromosomally integrated to reduce plasmid burden and improve pathway balance. Curcumin and ferulic acid production were evaluated alongside cell growth (OD_600_). (C) Effects of targeted gene deletions on metabolic flux redistribution. Deletion of *tyrR*, *crr*, *ptsG*, *pheA*, and *pykF* enhanced precursor supply and improved curcumin production. *tyrR*, HTH‐type transcriptional regulatory protein TyrR; *crr*, PTS system glucose‐specific *EIIA* component; *ptsG*, PTS system glucose‐specific EIICB component; *pheA*, bifunctional chorismate mutase/prephenate dehydratase; and *pykF*, pyruvate kinase I. (D) Orbitrap Eclipse Tribrid high‐resolution mass spectrometry‐assisted analysis of L‐DOPA oxidative degradation and melanin‐like pigment formation. (E) Identification of metabolic bottlenecks through supplementation of pathway intermediates (tyrosine, L‐DOPA, *p*‐coumaric acid, caffeic acid, and ferulic acid) in strain Cur59. Results are mean ± standard deviation (SD) from three biological replicates.

Efficient metabolic flux redistribution is essential for preparation of complex natural products with long biosynthetic pathways. To optimize curcumin biosynthesis, a HIDTE strategy was implemented in a previously constructed high‐producing strain [27]. The auxiliary modules *ribF*, which supports intracellular flavin cofactor supply, and *TcTAL*, an alternative tyrosine ammonia‐lyase introduced to balance aromatic acid flux, were selected for chromosomal integration. The resulting strain Cur53 produced 70.2 mg/L curcumin and exhibited improved growth compared with the parental strain Cur49, indicating that relocation of non‐essential modules reduced expression burden while maintaining pathway activity (Figure 1B). To further enhance precursor availability, targeted deletions were introduced to redirect carbon flux toward the phenylpropanoid branch. Deletion of *tyrR* relieved feedback repression of aromatic amino acid biosynthesis, whereas disruption of *crr* and *ptsG* increased PEP availability. Additional deletion of *pheA* and *pykF* further improved curcumin production, increasing the titer to 81.87 mg/L (Figure 1C). Evaluation of different replicon combinations showed that the f1 ori‐p15A ori configuration remained the optimal copy number arrangement (Figure S1).

Metabolic bottleneck analysis was then conducted by supplementing key intermediates in the curcumin biosynthetic pathway. Significant accumulation of L‐DOPA was observed, and supplementation with tyrosine partially improved curcumin production (Figure 1E), indicating that the tyrosine‐*p*‐coumaric acid/L‐DOPA‐caffeic acid node represents a critical metabolic bottleneck requiring further engineering. To further determine whether this bottleneck was associated with oxidative instability, we analyzed the oxidation rate of L‐DOPA and L‐DOPA‐derived oxidation products under oxidative culture conditions (Figure 1D). The annotated intermediates supported an oxygen‐dependent melanogenesis‐like degradation route, primarily involving DHI/DHICA‐related indolic intermediates and subsequent formation of melanin‐like pigments [28]. Furthermore, L‐DOPA consumption showed a positive linear correlation with A_400_ (Figure S2) [29], supporting the use of A_400_ as an operational index of oxidation‐associated browning associated with L‐DOPA consumption. Together, these results indicate that L‐DOPA accumulation is not merely a pathway‐flux bottleneck, but also an oxidation‐prone metabolic node that diverts carbon flux toward melanin‐like byproducts. This finding motivated subsequent flux‐protection engineering to enable low‐pH stabilization through acid adaptation, maintain reducing‐equivalent availability through respiratory‐chain modulation, and rebalance HpaB substrate preference away from L‐DOPA formation through enzyme engineering.

### 3.2 Adaptive Acid Evolution Remodels Nitrogen Flux and Improves Physiological Robustness

**FIGURE 2:**
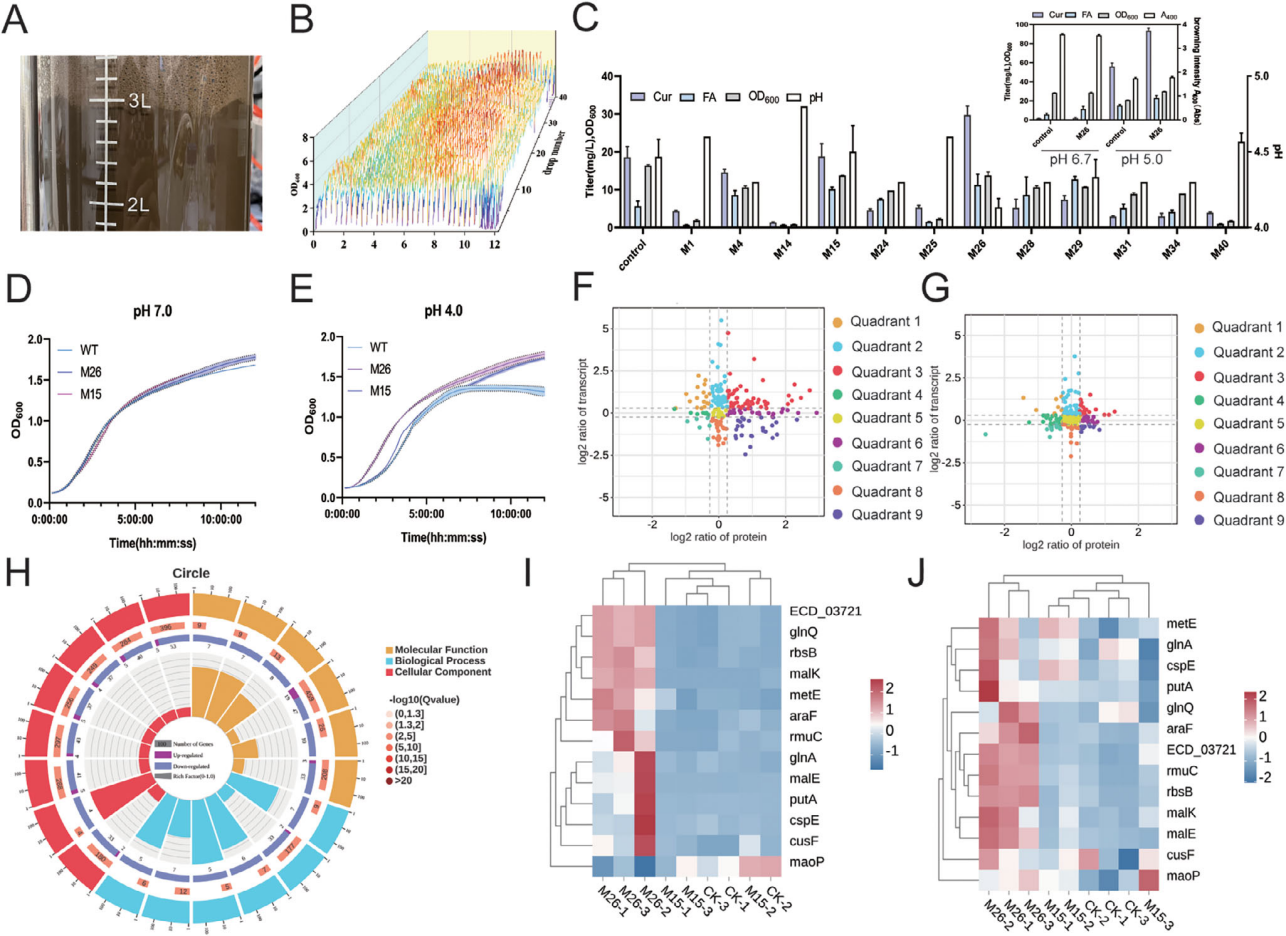
Adaptive acid tolerance evolution and multi‐omics characterization of acid‐resistant mutants. (A) Browning of culture medium caused by oxidative conversion of accumulated L‐DOPA during fermentation. (B) High‐throughput adaptive laboratory evolution under stepwise pH reduction (pH 7.0–4.0) using the MMC platform. (C) Production performance of evolved mutants under acidic fermentation conditions, including curcumin titer, ferulic acid accumulation, cell growth (OD_600_), and final pH. The inset shows the fermentation performance of the parental control strain and the acid‐evolved mutant M26 under different pH conditions, with A_400_ included as an oxidation‐associated browning index. (D,E) Growth curves of the parental strain (WT) and evolved mutants M15 and M26 under neutral (pH 7.0) and acidic (pH 4.0) conditions. (F and G) Integrated transcriptomic and proteomic correlation analysis of evolved strains relative to the control. Each point represents a gene or protein, and dashed lines indicate significance thresholds. (H) GO functional enrichment analysis of differentially expressed genes and proteins categorized by biological process, molecular function, and cellular component. (I,J) Heatmap visualization of representative differentially expressed genes and proteins involved in amino acid metabolism and transport pathways, highlighting metabolic reprogramming associated with acid tolerance. Results are mean ± SD from three independent biological replicates.

During fermentation, pronounced browning of the culture broth was observed, accompanied by substantial accumulation of L‐DOPA (Figure 2A), suggesting oxidative instability of this intermediate (Figure S3). To enhance cellular robustness under acidic conditions and potentially mitigate intermediate oxidation, adaptive acid tolerance evolution was performed using a MMC platform with stepwise pH reduction from 7.0 to 4.0 (Figure 2B and Figure S4) [30]. Twelve evolved mutants exhibiting improved growth were obtained and further evaluated for production performance. Among them, M15 and M26 retained both growth and production advantages under acidic fermentation conditions (Figure 2C). M15 produced 18.5 mg/L curcumin with OD_600_ = 16.4 at pH 4.47, while M26 reached 29.7 mg/L with OD_600_ = 13.8 at pH 4.10, compared with the control strain (18.8 mg/L, OD_600_ = 27.48, pH 5.13). Growth curve analysis further indicated that M26 exhibited a clear growth advantage during the latter stages, whereas M15 showed improved growth throughout cultivation. Moreover, comparison of pH 6.7 and pH 5.0 shake‐flask fermentations showed that M26 maintained superior production and growth under acidic conditions, reaching 93.3 mg/L curcumin with OD_600_ = 29.69 at pH 5.0 compared with the control strain (55.87 mg/L, OD_600_ = 26.81), while the A_400_‐based browning index was markedly lower at pH 5.0 than at pH 6.7 (Figure 2C).

Multi‐omics analysis showed that M26 underwent a more pronounced acid‐adaptive reprogramming than M15 at both the transcriptomic and proteomic levels (Figure 2F,G). In M26, coordinated changes in nitrogen and glutamate/glutamine metabolism, polyamine remodeling, and stress responses were associated with enhanced proton buffering and metabolic homeostasis, thereby supporting improved robustness under acidic conditions. (Figure 2I,J, Tables S3 and S4). By contrast, M15 exhibited a similar but much weaker adaptive trend. These results suggest that M26 acquired acid robustness to sustain production under low‐pH conditions favorable for reducing oxidation‐associated intermediate loss, supporting its selection as the chassis for subsequent engineering. The persistence of L‐DOPA under low‐pH conditions was consistent with reduced oxidative degradation, while its accumulation indicated continued carbon diversion into this off‐pathway branch. Subsequent flux rebalancing was therefore aimed at reducing L‐DOPA accumulation, thereby optimizing pathway flux and further lowering its overall oxidation rate. Therefore, the next stage of engineering focused on optimizing this oxidation‐sensitive metabolic node and reshaping intracellular electron allocation to reduce oxidative pressure and improve productive flux toward curcumin.

### 3.3 Respiratory Chain Modulation Reallocates Intracellular Electron Flux

**FIGURE 3:**
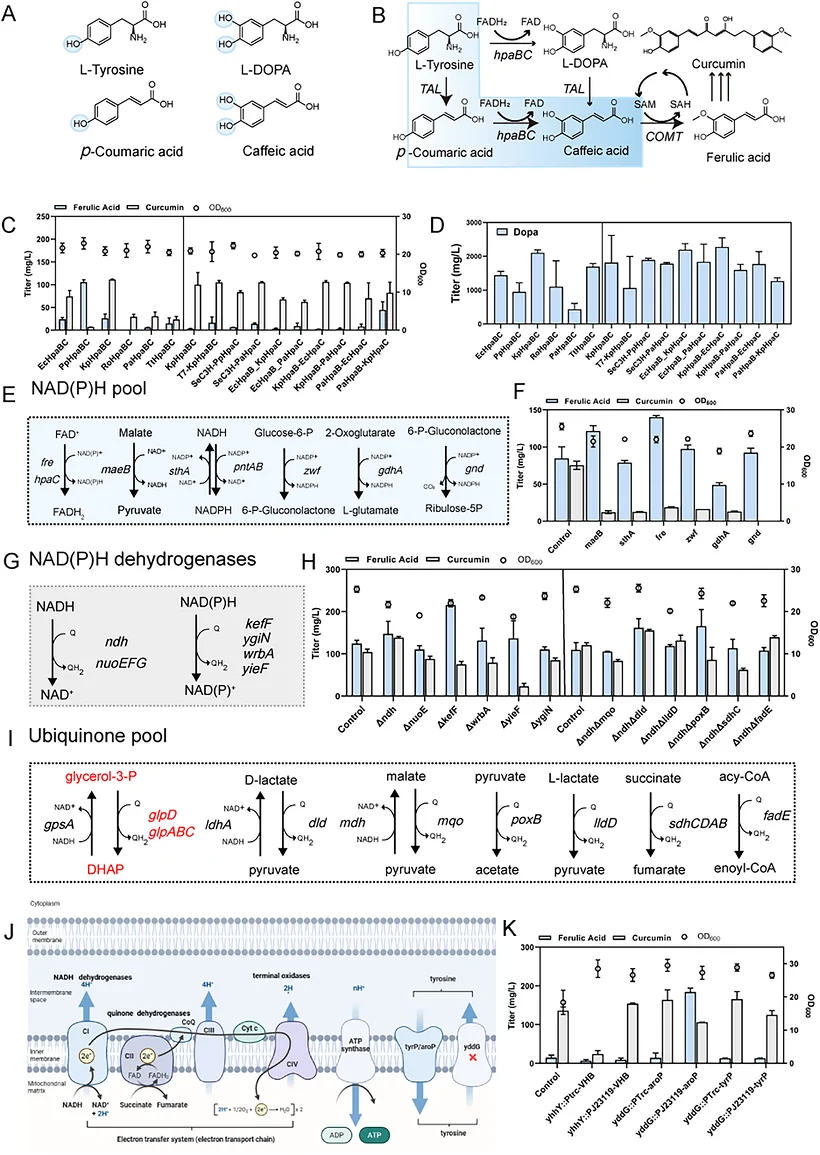
Optimization of oxidation‐prone upstream reactions and respiratory chain engineering to improve curcumin biosynthesis. (A) Chemical structures of key intermediates (L‐tyrosine, L‐DOPA, *p*‐coumaric acid, caffeic acid) in the curcumin pathway, highlighting the catechol moiety of L‐DOPA. (B) Schematic representation of upstream deamination and hydroxylation reactions catalyzed by TAL and HpaBC, highlighting flux redirection toward the *p*‐coumaric acid branch. (C,D) Curcumin titer and L‐DOPA accumulation in strains expressing HpaBC complexes from different sources. (E) NAD(P)H regeneration pathways targeted for cofactor engineering. (F) Effects of NAD(P)H pool enhancement on curcumin titer. (G) Overview of respiratory chain‐associated NAD(P)H dehydrogenases contributing to electron transfer to the quinone pool. (H) Evaluation of production performance in respiratory chain‐engineered strains. (I) Screening and deletion of quinone‐associated dehydrogenases to enhance quinone pool accumulation. (J) Schematic illustration of coupled respiratory chain engineering, VHb introduction, and tyrosine transport reconstruction for efficient curcumin biosynthesis under oxygen‐limited conditions. (K) Effects of the coupled strategy integrating respiratory chain engineering, VHb introduction, and tyrosine transport reconstruction on curcumin biosynthesis under oxygen‐limited conditions. Results are mean ± SD from three independent biological replicates.

Due to the presence of a catechol moiety, L‐DOPA exhibits higher redox reactivity than *p*‐coumaric acid and is therefore more susceptible to spontaneous oxidation under fermentation conditions (Figure 3A and Figure S5). To reduce the accumulation of oxidation‐prone intermediates and redirect metabolic flux toward *p*‐coumaric acid formation, upstream deamination and hydroxylation reactions catalyzed by TAL and HpaBC were systematically optimized (Figure 3B). TAL variants from different sources (*Fj*TAL, *Tc*TAL, and *Rg*TAL) and multiple HpaBC complexes (*Ec*HpaBC, *Tt*HpaBC, *Ro*HpaBC, *Pp*HpaBC, *Pa*HpaBC and *Kp*HpaBC) were evaluated. *Fj*TAL remained the most effective deaminase among the TAL candidates (Figure S6). Among the HpaBC variants, *Kp*HpaB resulted in the highest curcumin titer (111.3 mg/L). However, L‐DOPA accumulation increased to 46.3%, indicating that enhanced curcumin titer was likely associated with stronger hydroxylation activity rather than improved substrate discrimination toward *p*‐coumaric acid. Combinatorial pairing of different HpaB and HpaC subunits further confirmed that the native *Kp*HpaB complex remained the optimal configuration (Figure 3C,D). To promote conversion of accumulated L‐DOPA, enforced spatial colocalization of TAL and HpaB was attempted using the scaffold protein CipB. However, curcumin was not detected under this condition (Figure S7) [31], suggesting that steric interference may have impaired catalytic efficiency instead of facilitating substrate channeling.

Given that polyphenolic intermediates are vulnerable to oxidation under high DO conditions, redox redistribution was next explored under oxygen‐limited fermentation. Direct enlargement of the NAD(P)H pool by overexpressing NAD(P)H‐generating enzymes reduced curcumin titers (Figure 3E,F and Figure S5), suggesting that excessive redox perturbation disrupted metabolic balance. Therefore, a milder respiratory‐chain strategy was adopted to reallocate reducing equivalents while retaining glycerol‐dependent biomass formation (Figure 3G,J). Deletion of *ndh* increased curcumin production by 32.8% to 138.4 mg/L, and additional deletion of *lldD* further increased the titer to 155.3 mg/L (Figure 3H,I). Consistently, respiratory‐chain remodeling increased the NADH/(NADH + NAD^+^) ratio throughout fermentation, reaching 0.556 in Cur118 vs. 0.323 in Cur95 at 24 h, while ATP availability was maintained. Under equal‐biomass conditions, Cur118 also showed higher whole‐cell hydroxylation activity and caffeic acid formation, supporting enhanced HpaBC‐dependent hydroxylation capacity (Figure S8). Under oxygen‐limited conditions, VHb expression and tyrosine transport reconstruction improved biomass formation and curcumin production to 165.5 mg/L, likely reflecting combined effects on oxygen utilization, respiratory metabolism, oxygen‐dependent hydroxylation, and cellular fitness (Figure 3J,K).

### 3.4 Directed Evolution of *Fj*TAL and *Kp*HpaB Rebalances the Oxidation‐Sensitive Node

**FIGURE 4:**
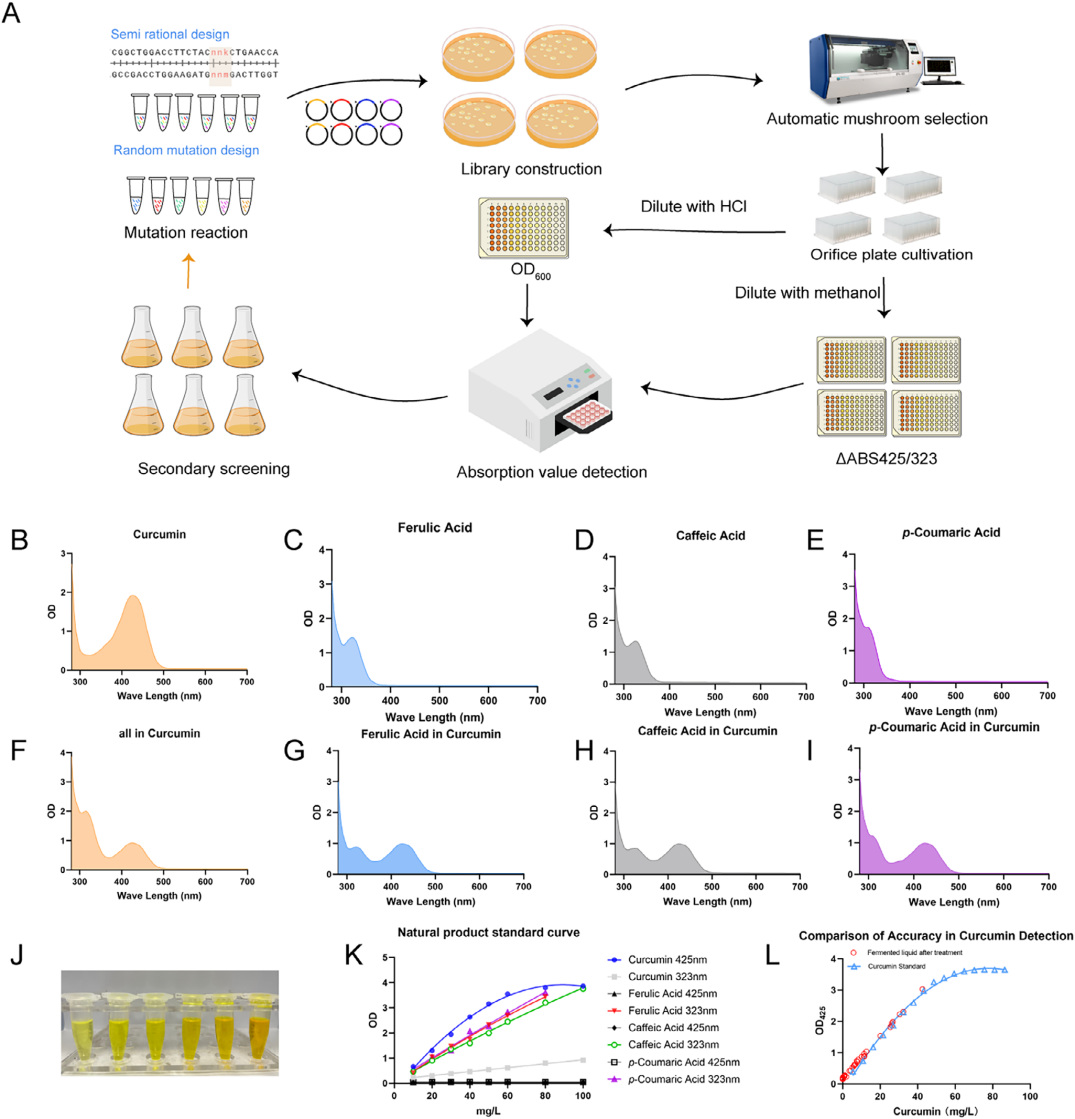
Construction and evaluation of a high‐throughput spectrophotometric method for curcumin and related phenolic compounds. (A) Schematic workflow of the high‐throughput screening platform integrating mutagenesis, library construction, automated colony selection, microplate cultivation, and absorbance‐based detection. (B–E) Full‐wavelength UV/Vis absorption spectra of curcumin (B), ferulic acid (C), caffeic acid (D), and *p*‐coumaric acid (E). (F) Full‐wavelength absorption spectrum of a mixture containing curcumin, ferulic acid, caffeic acid, and *p*‐coumaric acid. (G–I) Full‐wavelength absorption spectra of binary mixtures of curcumin with ferulic acid (G), caffeic acid (H), and *p*‐coumaric acid (I). (J) Visual color gradient of curcumin dissolved in methanol at increasing concentrations from 10 to 1000 mg/L. (K) Standard curves of curcumin, ferulic acid, caffeic acid, and *p*‐coumaric acid measured using a microplate reader at characteristic wavelengths. (L) Accuracy evaluation of the high‐throughput spectrophotometric method compared with HPLC analysis for quantification of fermentation products. Results are mean ± SD from three independent experiments.

**FIGURE 5:**
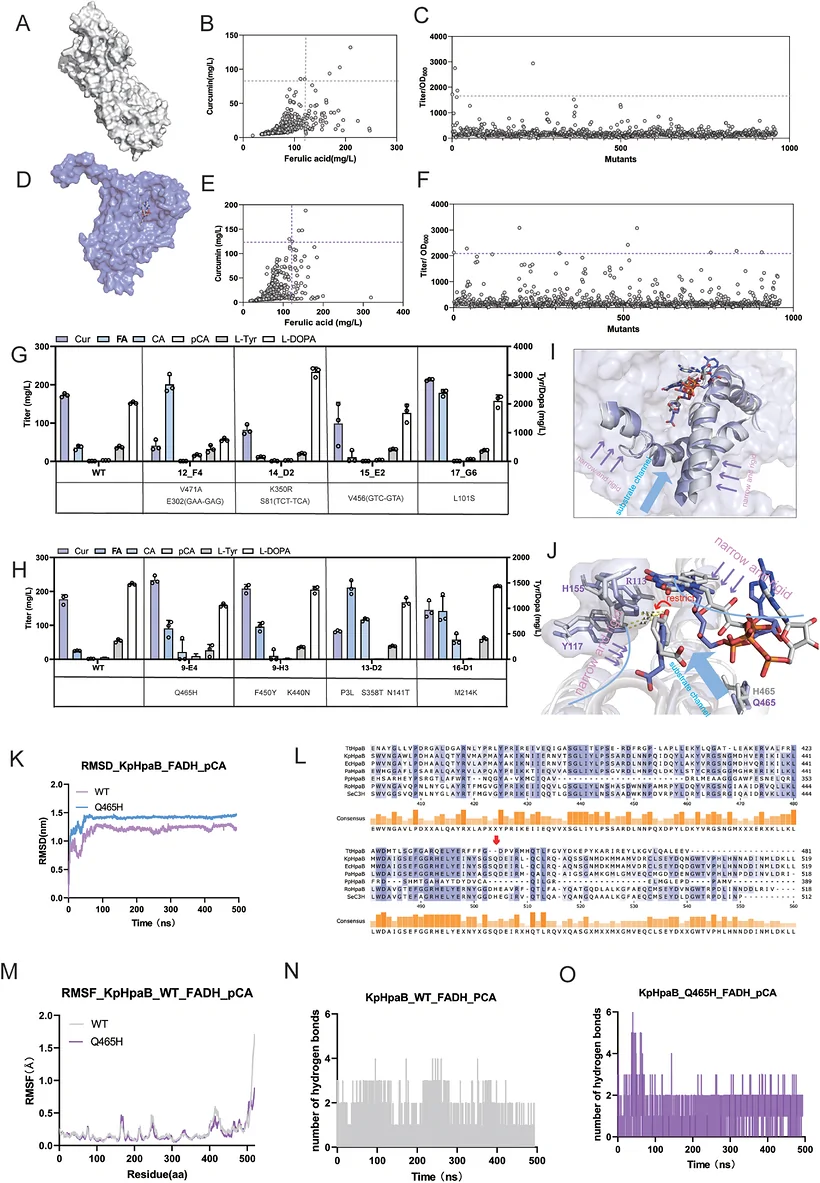
Directed evolution of key rate limiting enzymes. (A) Molecular docking model of *Fj*TAL in complex with L‐tyrosine. (B) Fermentation performance of the *Fj*TAL mutant library, showing the relationship between ferulic acid and curcumin titers. (C) Distribution of curcumin titer normalized by OD_600_ in the *Fj*TAL mutant library. (D) Molecular docking model of *Kp*HpaB in complex with FADH and *p*‐coumaric acid. (E) Fermentation performance of the *Kp*HpaB mutant library, illustrating the correlation between ferulic acid titer and curcumin titer. (F) Distribution of curcumin titer normalized by OD_600_ in the *Kp*HpaB mutant library. In all panels, horizontal and vertical dashed lines indicate the WT reference. (G) Shake‐flask validation of selected *Fj*TAL mutants showing titers of curcumin (Cur), ferulic acid (FA), caffeic acid (CA), *p*‐coumaric acid (pCA), L‐tyrosine (L‐Tyr), and L‐DOPA. (H) Shake‐flask validation of selected *Kp*HpaB mutants, showing titers of Cur, FA, CA, pCA, L‐Tyr, and L‐DOPA. (I) Structural superposition of *Kp*HpaB^Q465H^ and WT *Kp*HpaB derived from dynamic modeling, showing that the Q465H substitution narrows the substrate access channel. (J) Detailed view of the binding conformations of *p*‐Coumaric acid and FADH within the catalytic pocket of *Kp*HpaB^Q465H^ and *Kp*HpaB^WT^, highlighting the altered pocket geometry and local interaction network caused by the mutation. (K) RMSD analysis of *Kp*HpaB^Q465H^ and *Kp*HpaB^WT^ during molecular dynamics simulations. (L) Multiple sequence alignment of HpaB homologs around residue 465. (M) RMSF analysis of *Kp*HpaB^Q465H^ and *Kp*HpaB^WT^ during molecular dynamics simulations. (N) Hydrogen‐bond occupancy profile of *p*‐Coumaric acid in the active pocket of *Kp*HpaB^WT^ during molecular dynamics simulations. (O) Hydrogen bond occupancy profile of *p*‐Coumaric acid in the active pocket of *Kp*HpaB^Q465H^ during molecular dynamics simulations. Results are mean ± SD from three independent biological replicates.

Despite previous engineering strategies having enhanced carbon flux toward curcumin biosynthesis, substantial accumulation of L‐DOPA persisted. To rebalance flux at key metabolic nodes, a high‐throughput screening platform was established for rapid evaluation of *Fj*TAL and *Kp*HpaB mutant libraries, together with quantitative detection methods for curcumin and ferulic acid (Figure 4A–L). Using curcumin titer and Cur/OD_600_ as dual screening criteria, 3324 mutants were screened (Figure 5B,C,E,F and Figure S13). Among them, eight promising variants were selected for sequencing and secondary validation, followed by shake‐flask re‐evaluation of representative high‐performing mutants. *Fj*TAL^L101S^ increased curcumin titer by 20.5%, but also elevated L‐DOPA accumulation by 24.6% and reduced tyrosine level by 10.4% (Figure 5G), indicating enhanced deamination activity without effective optimization of substrate preference. In contrast, *Kp*HpaB^Q465H^ increased curcumin titer by 35.4%, while lowering L‐DOPA accumulation by 28.3% and decreasing tyrosine level by 35.1%, suggesting improved substrate recognition and more balanced flux distribution. By comparison, *Kp*HpaB^F450Y/K440N^ decreased tyrosine concentration by 35.1% but led to a 29.1% increase in L‐DOPA accumulation (Figure 5H), indicating an enhanced tendency toward tyrosine hydroxylation but no improvement in overall flux balance, thereby resulting in further intermediate accumulation. However, iterative validation of the above mutants revealed adverse effects, further confirming that single‐round improvements did not necessarily translate into robust combinatorial performance (Figure S11). Consistently, purified‐enzyme kinetics showed that *Kp*HpaB^Q465H^ improved catalytic efficiency toward *p*‐coumaric acid but markedly reduced that toward L‐tyrosine (Figure S10 and Table S5), supporting enzyme‐level flux balancing that favored productive caffeic acid formation over L‐DOPA accumulation.

Further analysis showed that residue 465 displayed a Q/H distribution tendency among *Kp*HpaB homologs from different sources. This site is located at the C‐terminal end of a key α‐helix and the beginning of the adjacent loop region, where it may influence local conformational flexibility and substrate channel geometry, thereby affecting substrate entry and active site preorganization (Figure 5I,L). Molecular dynamics simulations showed that KpHpaB^Q465H^ exhibited altered conformational dynamics relative to the WT enzyme, as reflected by the RMSD and localized RMSF changes in channel‐associated regions (Figure 5K,M). Structural analysis further revealed rearrangement of the local interaction network surrounding *p‐*coumaric acid in the mutant binding pocket (Figure 5J). Consistently, *p*‐coumaric acid formed a more extensive and persistent hydrogen‐bond network in KpHpaB^Q465H^ than in the WT enzyme (Figure 5N,O), supporting enhanced stabilization of the productive substrate‐binding state. Together with the pH‐dependent kinetics showing increased activity toward *p*‐coumaric acid but reduced activity toward L‐tyrosine across pH 5.7–7.5 (Figure S10) and the altered Gibbs free‐energy landscape (Figure S14), these results suggest that Q465H remodels conformational dynamics and substrate‐binding geometry to shift catalytic preference toward *p*‐coumaric acid, thereby reducing L‐DOPA formation and improving flux.

### 3.5 PH–DO‐Guided Curcumin Scale‐up and Caffeic Acid Validation of Flux Protection

**FIGURE 6:**
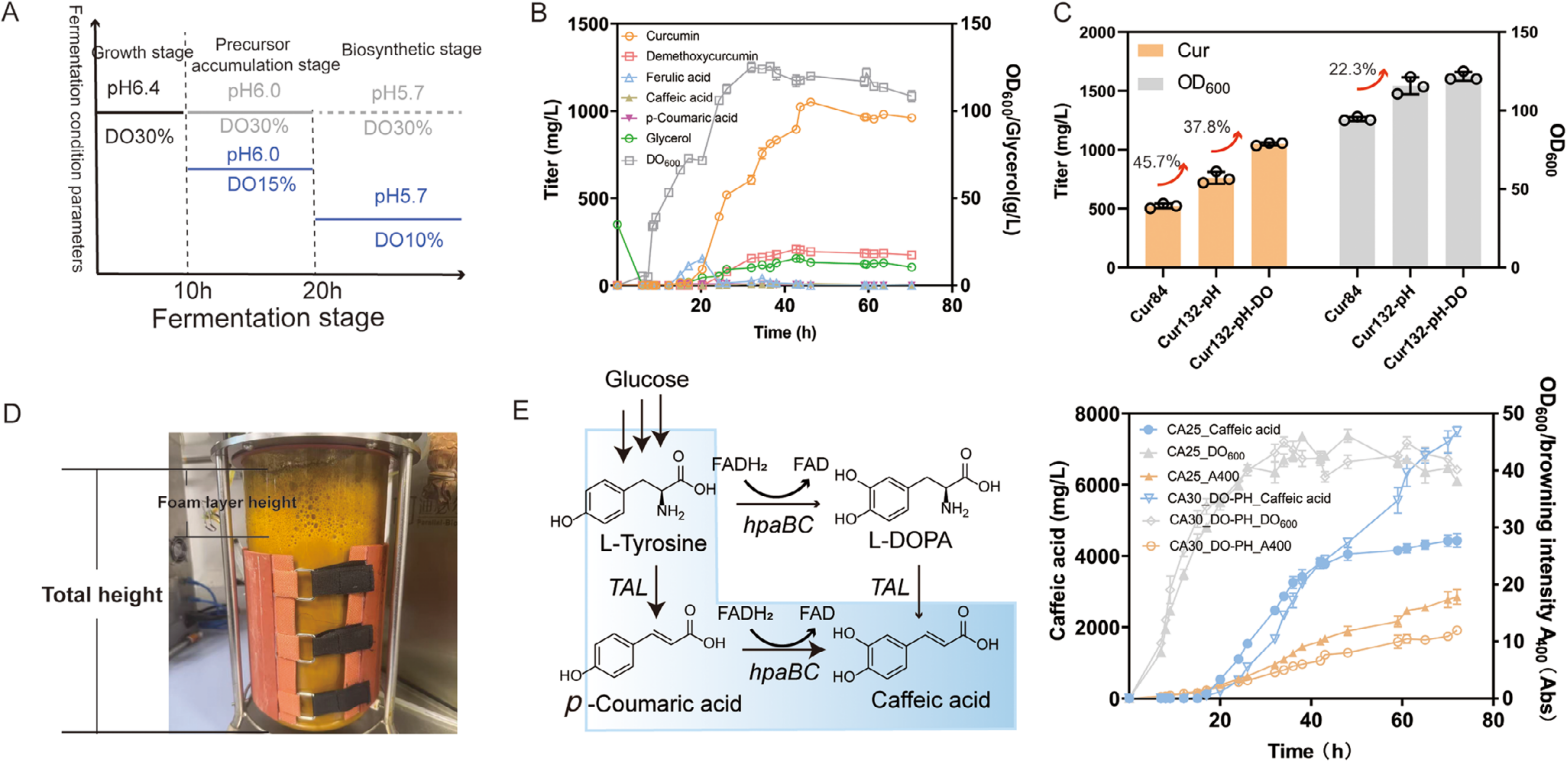
Multistage pH‐DO coordinated control enhances curcumin titer in a 5 L fermentation bioreactor. (A) Schematic illustration of the multistage fermentation strategy. The process is divided into three phases: growth stage (pH 6.4, 30% DO), precursor accumulation stage (pH 6.0, 15% DO), and biosynthetic stage (pH 5.7, 10% DO). (B) Fermentation performance of strain Cur132 under the coordinated pH‐DO multistage control strategy. Curcumin, demethoxycurcumin, ferulic acid, caffeic acid, *p*‐coumaric acid, glycerol consumption, and cell growth (OD_600_) were monitored over time. Compared with the pH‐only strategy, coordinated pH‐DO control accelerated curcumin accumulation and increased final titers. (C) Comparison of curcumin titers and biomass among engineered strains under 5 L fermentation bioreactor conditions. (D) Representative image of the 5 L bioreactor during fermentation, showing foam formation and liquid height, highlighting oxygen transfer limitations during scale‐up. from three independent experiments. (E) Extension of the integrated flux‐protection framework to caffeic acid production in a 5 L bioreactor using the same coordinated pH–DO multistage control strategy as that applied for curcumin fermentation. Results are mean ± SD from three independent biological replicates.

Oxidative browning of L‐DOPA during fermentation (Figure 2A) caused substantial carbon flux dissipation, while persistent foaming in the 5 L bioreactor impaired oxygen transfer and created a coupled low‐pH and oxygen‐limited environment. To improve production robustness under scale‐up conditions, the engineered strain developed through acid tolerance evolution, respiratory chain modulation, oxygen‐coupling enhancement, and enzyme engineering was evaluated using a multistage pH–DO control strategy (Figure 6A). Under the pH single‐factor multistage strategy, Cur132 reached 762.7 mg/L curcumin at 72 h, with 22.3% higher biomass and 45.7% higher curcumin production than Cur84 (Figure S15). When coordinated pH–DO control was applied, Cur132 produced 1075.2 mg/L curcumin and 200.17 mg/L demethoxycurcumin at 46 h (Figure 6B,C). Compared with the pH‐only strategy, biomass increased by an additional 5.2%, curcumin production improved by 37.8%, and fermentation time was markedly shortened. Consistently, the background‐corrected A_400_ progressively decreased from Cur84 to Cur132‐pH and Cur132‐pH–DO, while FPI increased from 102.76 to 232.81 and 1320.88, with corresponding rFPI_i_ values of 2.27 and 5.67 (Table 1). These results indicate that the integrated strain–process strategy improved productive output while reducing oxidation‐associated carbon dissipation during scale‐up fermentation.

To evaluate the transferability of the flux‐protection strategy within a related HpaB‐dependent phenylpropanoid pathway, a stepwise caffeic acid‐producing strain series (CA24–CA30) was constructed. Caffeic acid production increased from 382.41 to 850.40 mg/L, whereas background‐corrected A_400_ decreased from 7.392 to 4.619. Accordingly, FPI increased from 51.73 to 184.11, with rFPI_i_ values of 1.22, 1.09, 1.64, 1.29, 1.06 and 1.18 following acid adaptation, *Kp*HpaBC introduction, Q465H substitution, Δ*ndh*/Δ*lldD*‐mediated respiratory‐chain remodeling, VHb expression and transporter engineering, respectively. Because HpaB can also hydroxylate L‐tyrosine to oxidation‐prone L‐DOPA, L‐DOPA, rather than the desired terminal product caffeic acid, was defined as the principal off‐pathway carbon‐leakage node. At the 5 L scale, caffeic acid production increased from 4.44 g/L in CA25 under the original process to 6.24 g/L in CA30 under the same process and further to 7.50 g/L under multistage pH–DO control, while A_400_ decreased from 17.83 to 15.00 and 11.97, respectively (Figure 6E and Figure S15). Correspondingly, FPI increased from 248.81 to 415.73 and 626.64, with rFPI_i_ values of 1.67 and 1.51 (Table 1). As the final comparison incorporated both strain engineering and process optimization, it was interpreted as an integrated strain–process validation. Collectively, these results support the transferability of the major engineering principles within a related HpaB‐dependent phenylpropanoid pathway.

Product titers and A_400_ values are presented as mean ± SD from three independent biological replicates. A_400_ represents the absorbance of cell‐free fermentation supernatants at 400 nm and was used as an operational index of oxidation‐associated browning. FPI was calculated independently for each biological replicate as the ratio of product titer to the corresponding A_400_ value, representing product output relative to oxidation‐associated browning. The relative FPI (rFPI_i_) represents the sequential change in FPI between adjacent strains or process conditions and is used only as a descriptive comparison rather than as a measure of the independent contribution of a specific engineering layer. The relative FPI (rFPI_i_) reflects the sequential change in FPI between adjacent engineering steps and is used only as a descriptive comparison rather than as a measure of the independent anti‐oxidation contribution of a specific engineering layer. Results are mean ± SD from three independent experiments.

## 4 Discussion

Oxidation‐driven instability is an important constraint limiting the efficient biosynthesis of polyphenolic compounds, yet it has received less attention than conventional flux optimization. In this study, L‐DOPA was identified as an oxidation‐prone carbon‐leakage node in engineered curcumin‐producing cells, supporting a reframing of curcumin biosynthesis from flux amplification to flux protection. DHI/DHICA‐related intermediates and the positive correlation between L‐DOPA consumption and A_400_ supported an oxygen‐dependent melanogenesis‐like degradation route. Because multiple pathway and matrix components can contribute to absorbance at 400 nm, A_400_ was therefore interpreted as an operational index of oxidation‐associated browning rather than a compound‐specific measure of L‐DOPA oxidation. Guided by this concept, we developed a hierarchical engineering strategy integrating metabolic flux redistribution, redox balancing, host robustness enhancement, and process control, thereby reducing oxidative carbon dissipation and improving pathway efficiency. As a result, the engineered strain achieved 1075.2 mg/L curcumin in a 5 L bioreactor, demonstrating that efficient production in redox‐sensitive pathways depends not only on catalytic capacity but also on protection of productive carbon flux. Not all layers directly suppressed oxidation; *Kp*HpaBC, VHb, and transport engineering mainly supported pathway performance, whereas acid adaptation, respiratory‐chain remodeling, Q465H, and pH–DO control more directly protected productive flux.

Acid adaptation enables host robustness under low‐pH conditions that retard L‐DOPA auto‐oxidation and help preserve productive carbon flux. Previous studies showed that improved microbial acid tolerance is often achieved through reinforcement of a single canonical acid resistance module, such as glutamate‐dependent acid resistance [32], enhanced proton‐consuming reactions [33], or transporter‐mediated relief of acid burden [34]. In contrast, the acid‐tolerant phenotype obtained in this study appears to arise from coordinated physiological reprogramming. ALE‐derived acid‐adapted *E. coli* cells reportedly undergo system‐level metabolic rewiring, and acid stress is alleviated through transporter‐related functions [35]. However, the phenotype observed here appears to extend beyond these individual features. Specifically, the acid‐adapted state in our strain integrates nitrogen metabolic reprogramming, enhanced proton‐consuming capacity, and upregulation of organic acid efflux‐related functions. Collectively, these results suggest a more multidimensional and coordinated adaptive pattern underlying acid tolerance in the present study. For curcumin biosynthesis, this systems‐level adaptation is particularly valuable because it not only improves acid tolerance, but also suppresses phenolic intermediate oxidation and preserves productive carbon flux. Thus, acid adaptation links host robustness with pathway stabilization and may facilitate flux protection in related HpaB‐dependent phenylpropanoid pathways under acidic production conditions.

Respiratory chain remodeling improves flux protection under oxygen‐limited conditions. Low oxygen restricts electron transfer and cofactor turnover, thereby reducing the effective availability of reducing equivalents for growth and target biosynthesis [22]. Previous studies showed that strengthening NAD(P)H regeneration can relieve redox pressure in some systems, but often at the expense of central metabolic balance, particularly in pathways involving oxygen‐dependent reactions [30, 36]. This conclusion was corroborated in the present work. In this context, respiratory chain remodeling represents a milder and more effective strategy, as it redistributes electron flux to support biosynthesis while stabilizing oxidation‐prone metabolic nodes [37]. Deletion of *ndh* and *lldD* likely reduced futile electron dissipation and improved quinone‐associated electron partitioning. Moreover, VHb can provide low‐oxygen compensation by improving oxygen capture and utilization, thereby supporting respiration and oxygen‐dependent hydroxylation without increasing bulk oxygen exposure [38]. This supports its use as an oxygen coupling module following respiratory chain remodeling, while the resulting phenotype further suggests that oxygen limitation was alleviated in the engineered strain. Overall, compared with simply enhancing NAD(P)H regeneration or increasing DO, this strategy places greater emphasis on coupling electron routing with process demand, and is therefore better suited for stabilizing biosynthetic pathways containing oxidation‐prone intermediates.

Flux‐matched enzyme optimization is more effective than activity‐centered engineering for stabilizing oxidation‐prone metabolic nodes. Because colony‐color‐based screening limits quantitative mutant evaluation [39, 40], we developed a more analytically precise high‐throughput screening method to identify flux‐matched variants. Unlike HpaB^G295R^, which mainly improved tyrosine hydroxylation activity [11], *Kp*HpaB^Q465H^ rebalanced substrate preference at the L‐DOPA node, redirecting flux toward the productive branch. This enzyme‐level balancing was further coupled with pH–DO coordinated fermentation, which is critical for maintaining precursor accumulation, redox homeostasis, and product formation during scale‐up [21, 23]. The integrated strategy enabled 1075.2 mg/L curcumin at 46 h in a 5 L bioreactor, with a productivity of 23.4 mg·L^−^^1^·h^−^^1^, an apparent glycerol‐based yield of ∼ 0.010 g/g, and a carbon‐molar conversion of ∼ 1.8%. Compared with the reported 978 mg/L co‐culture process, the single‐strain system increased titer by 9.9% and productivity by 72.1% while shortening fermentation time by 36.1%. Transfer to the related HpaB‐dependent caffeic acid pathway yielded 7.50 g/L with reduced A_400‐_associated browning, approaching the previously reported 7.9 g/L and remaining below the current record of 9.61 g/L. This validation supports transferability within related HpaB‐dependent phenylpropanoid pathways. Flux protection is most applicable to pathways containing oxidation‐prone phenolic intermediates and requires pathway‐specific optimization. Curcuminoid profiling identified 200.17 mg/L demethoxycurcumin (15.7%), whereas bisdemethoxycurcumin was not detected (Figure S16), indicating that further optimization is required for high‐purity curcumin production [41].

## 5 Conclusions

Unlike conventional strategies that focus on isolated pathway bottlenecks, this study addressed oxidative carbon dissipation as a system‐level constraint in microbial polyphenol biosynthesis. By resolving L‐DOPA as an oxidation‐prone carbon‐leakage node, we established a hierarchical flux‐protection framework that integrates pathway flux redistribution, host adaptation, respiratory chain remodeling, enzyme‐level flux matching, and process‐level pH–DO control. This integrated strategy minimized oxidation‐associated carbon loss while maintaining cellular robustness, enabling 1075.2 mg/L curcumin production in a 5 L bioreactor, the highest reported to date. Transfer of selected engineering principles to the related HpaB‐dependent caffeic acid pathway yielded 7.50 g/L with reduced A_400_‐associated browning, supporting pathway‐context‐specific transferability. Although further optimization of product‐spectrum control and downstream purification will be required for high‐purity curcumin production, this work establishes flux protection as an effective strategy for curcumin biosynthesis and supports the potential transferability of selected engineering principles to related HpaB‐dependent phenylpropanoid pathways.

## Author Contributions

**Jianbin Chen**: investigation, visualization, writing – original draft, writing – review and editing. **Qihang Chen**: investigation. **Shurong Zhang**: investigation. **Yajuan Su**: investigation. **Xiaoyi Zou**: investigation. **Ke Wang**: investigation. **Shike Liu**: investigation. **Qian He**: investigation. **Liang Zhang**: investigation. **Weizhu Zeng**: investigation. **Jingwen Zhou**: supervision, funding acquisition, writing – review and editing.

## Conflicts of Interest

The authors declare no conflicts of interest.

## Supporting information




**Supporting File**: advs77628‐sup‐0001‐SuppMat.docx.

## Data Availability

The data that support the findings of this study are available from the corresponding author upon reasonable request.
