# Supplementary material for: Hierarchical Flux‐Protection Engineering Stabilizes Oxidation‐Prone Metabolic Nodes for High‐Level Curcumin Biosynthesis
Source: Adv Sci (Weinh). 2026 Sep 13:e77628. Online ahead of print. doi: 10.1002/advs.77628 (PMC13572824; doi:10.1002/advs.77628)
Supplement: Supplementary file 1 [file ADVS-9999-e77628-s001.docx]

**Hierarchical flux-protection engineering stabilizes oxidation-prone metabolic nodes for high-level curcumin biosynthesis**

Jianbin Chen^1,2^, Qihang Chen^1,2^, Shurong Zhang^1,2^, Yajuan Su^1,2^, Xiaoyi Zou^1,2^,Ke Wang, Shike Liu^1,2^, Qian He^1,2^, Liang Zhang^1^, Weizhu Zeng^1,2,3^, Jingwen Zhou^1,2,3^^*^

^1^ Engineering Research Center of Ministry of Education on Food Synthetic Biotechnology, Jiangnan University, 1800 Lihu Road, Wuxi, Jiangsu 214122, China;

^2^ Science Center for Future Foods, Jiangnan University, 1800 Lihu Road, Wuxi, Jiangsu 214122, China;

^3^ Jiangsu Province Engineering Research Center of Food Synthetic Biotechnology,

Jiangnan University, Wuxi 214122, China.

* Correspondence to:

Jingwen Zhou

Science Center for Future Foods, Jiangnan University, 1800 Lihu Rd, Wuxi, Jiangsu 214122, China.

Phone: +86-510-85918310, Fax: +86-510-85918310

E-mail: zhoujw1982@jiangnan.edu.cn.

# Supplementary Tables

## Table S1. Strains used in this study.

| **Strains** | **Description** | **Sources** |
| --- | --- | --- |
| *E. coli* BL21(DE3) | Parent strain | Invitrogen |
| *E. coli* JM109 | Gene cloning | Invitrogen |
| *E. coli* BL21(DE3) Δ*tyrR*Δ*ptsG*Δ*crr*Δ*pheA* | *E. coli* BL21(DE3) with deletion of *tyrR,ptsG,crr* and *pheA* | Lab stock ^1^ |
| Cur49 | *E. coli* BL21(DE3) Δ*curA::srpB* Δ*tyrR*Δ*ptsG*Δ*crr*Δ*pheA*Δ*fadR*Δ*mcrC*, pCDF-T7-*TAL*-T7-*ribF* and pACYC-T7-*FjTAL*-*HpaBC*-T7-*AtCOMT* and pETDuet-T7-*aroG^fbr^*-*tyrC*-T7-*4CL*-*DCS*-(GGGGS)_3_-*CURS1* | Lab stock ^2^ |
| Cur50 | *E. coli* BL21(DE3) Δ*curA::srpB* Δ*tyrR*Δ*ptsG*Δ*crr*Δ*pheA*Δ*fadR*Δ*mcrC*, pACYC-T7-*FjTAL*-*HpaBC*-T7-*AtCOMT* and pETDuet-T7-*aroG^fbr^*-*tyrC*-T7-*4CL*-*DCS*-(GGGGS)_3_-*CURS1* | This study |
| Cur 51 | *E. coli* BL21(DE3) Δ*ushA::*T7*-ribF*Δ*curA::srpB* Δ*tyrR*Δ*ptsG*Δ*crr*Δ*pheA*Δ*fadR*Δ*mcrC*, pACYC-T7-*FjTAL*-*HpaBC*-T7-*AtCOMT* and pETDuet-T7-*aroG^fbr^*-*tyrC*-T7-*4CL*-*DCS*-(GGGGS)_3_-*CURS1* | This study |
| Cur 52 | *E. coli* BL21(DE3) Δ*ushA::*T7*-TcTAL*Δ*curA::srpB* Δ*tyrR*Δ*ptsG*Δ*crr*Δ*pheA*Δ*fadR*Δ*mcrC*, pACYC-T7-*FjTAL*-*HpaBC*-T7-*AtCOMT* and pETDuet-T7-*aroG^fbr^*-*tyrC*-T7-*4CL*-*DCS*-(GGGGS)_3_-*CURS1* | This study |
| Cur 53 | *E. coli* BL21(DE3) Δ*ushA::*T7*-ribF-TcTAL*Δ*curA::srpB*Δ*tyrR*Δ*ptsG*Δ*crr*Δ*pheA*Δ*fadR*Δ*mcrC*, pACYC-T7-*FjTAL*-*HpaBC*-T7-*AtCOMT* and pETDuet-T7-*aroG^fbr^*-*tyrC*-T7-*4CL*-*DCS*-(GGGGS)_3_-*CURS1* | This study |
| Cur 54 | *E. coli* BL21(DE3) Δ*ushA::*T7*-ribF-TcTAL*Δ*curA::srpB*Δ*fadR*Δ*mcrC*, pACYC-T7-*FjTAL*-*HpaBC*-T7-*AtCOMT* and pETDuet-T7-*aroG^fbr^*-*tyrC*-T7-*4CL*-*DCS*-(GGGGS)_3_-*CURS1* | This study |
| Cur55 | *E. coli* BL21(DE3) Δ*ushA::*T7*-ribF-TcTAL*Δ*curA::srpB*Δ*fadR*Δ*mcrC*Δ*tyrR*Δ*pheA*, pACYC-T7-*FjTAL*-*HpaBC*-T7-*AtCOMT* and pETDuet-T7-*aroG^fbr^*-*tyrC*-T7-*4CL*-*DCS*-(GGGGS)_3_-*CURS1* | This study |
| Cur56 | *E. coli* BL21(DE3) Δ*ushA::*T7*-ribF-TcTAL*Δ*curA::srpB*Δ*fadR*Δ*mcrC*Δ*tyrR*Δ*ptsG*Δ*crr* pACYC-T7-*FjTAL*-*HpaBC*-T7-*AtCOMT* and pETDuet-T7-*aroG^fbr^*-*tyrC*-T7-*4CL*-*DCS*-(GGGGS)_3_-*CURS1* | This study |
| Cur57 | *E. coli* BL21(DE3) Δ*ushA::*T7*-ribF-TcTAL*Δ*curA::srpB*Δ*fadR*Δ*mcrC*Δ*tyrR*Δ*ptsG*Δ*crr*Δ*pheA* pACYC-T7-*FjTAL*-*HpaBC*-T7-*AtCOMT* and pETDuet-T7-*aroG^fbr^*-*tyrA^fbr^*-T7-*4CL*-*DCS*-(GGGGS)_3_-*CURS1* | This study |
| Cur58 | *E. coli* BL21(DE3) Δ*ushA::*T7*-ribF-TcTAL*Δ*curA::srpB*Δ*fadR*Δ*mcrC*Δ*tyrR*Δ*ptsG*Δ*crr*Δ*pheA*Δ*ptsG::*T7-*glk*, pACYC-T7-*FjTAL*-*HpaBC*-T7-*AtCOMT* and pETDuet-T7-*aroG^fbr^*-*tyrC*-T7-*4CL*-*DCS*-(GGGGS)_3_-*CURS1* | This study |
| Cur59 | *E. coli* BL21(DE3) Δ*pykF*Δ*ushA::*T7*-ribF-TcTAL*Δ*curA::srpB*Δ*fadR*Δ*mcrC*Δ*tyrR*Δ*ptsG*Δ*crr*Δ*pheA*, pACYC-T7-*FjTAL*-*HpaBC*-T7-*AtCOMT* and pETDuet-T7-*aroG^fbr^*-*tyrC*-T7-*4CL*-*DCS*-(GGGGS)_3_-*CURS1* | This study |
| Cur60 | *E. coli* BL21(DE3) Δ*pykF::*T7*-ppsA*Δ*ushA::*T7*-ribF-TcTAL*Δ*curA::srpB*Δ*fadR*Δ*mcrC*Δ*tyrR*Δ*ptsG*Δ*crr*Δ*pheA*, pACYC-T7-*FjTAL*-*HpaBC*-T7-*AtCOMT* and pETDuet-T7-*aroG^fbr^*-*tyrC*-T7-*4CL*-*DCS*-(GGGGS)_3_-*CURS1* | This study |
| Cur61 | *E. coli* BL21(DE3) Δ*pykF*Δ*ushA::*T7*-ribF-TcTAL*Δ*curA::srpB*Δ*fadR*Δ*mcrC*Δ*tyrR*Δ*ptsG*Δ*crr*Δ*pheA*, pACYCDuet-T7-*FjTAL*-*HpaBC*-T7-*AtCOMT* and pCDFDuet-T7-*aroG^fbr^*-*tyrC*-T7-*4CL*-*DCS*-(GGGGS)_3_-*CURS1* | This study |
| Cur62 | *E. coli* BL21(DE3) Δ*pykF*Δ*ushA::*T7*-ribF-TcTAL*Δ*curA::srpB*Δ*fadR*Δ*mcrC*Δ*tyrR*Δ*ptsG*Δ*crr*Δ*pheA*, pACYCDuet-T7-*FjTAL*-*HpaBC*-T7-*AtCOMT* and pRSFDuet-T7-*aroG^fbr^*-*tyrC*-T7-*4CL*-*DCS*-(GGGGS)_3_-*CURS1* | This study |
| Cur63 | *E. coli* BL21(DE3) Δ*pykF*Δ*ushA::*T7*-ribF-TcTAL*Δ*curA::srpB*Δ*fadR*Δ*mcrC*Δ*tyrR*Δ*ptsG*Δ*crr*Δ*pheA*, pCDFDuet-T7-*FjTAL*-*HpaBC*-T7-*AtCOMT* and pACYCDuet-T7-*aroG^fbr^*-*tyrC*-T7-*4CL*-*DCS*-(GGGGS)_3_-*CURS1* | This study |
| Cur64 | *E. coli* BL21(DE3) Δ*pykF*Δ*ushA::*T7*-ribF-TcTAL*Δ*curA::srpB*Δ*fadR*Δ*mcrC*Δ*tyrR*Δ*ptsG*Δ*crr*Δ*pheA*, pCDFDuet-T7-*FjTAL*-*HpaBC*-T7-*AtCOMT* and pETDuet-T7-*aroG^fbr^*-*tyrC*-T7-*4CL*-*DCS*-(GGGGS)_3_-*CURS1* | This study |
| Cur65 | *E. coli* BL21(DE3) Δ*pykF*Δ*ushA::*T7*-ribF-TcTAL*Δ*curA::srpB*Δ*fadR*Δ*mcrC*Δ*tyrR*Δ*ptsG*Δ*crr*Δ*pheA*, pCDFDuet-T7-*FjTAL*-*HpaBC*-T7-*AtCOMT* and pRSFDuet-T7-*aroG^fbr^*-*tyrC*-T7-*4CL*-*DCS*-(GGGGS)_3_-*CURS1* | This study |
| Cur66 | *E. coli* BL21(DE3) Δ*pykF*Δ*ushA::*T7*-ribF-TcTAL*Δ*curA::srpB*Δ*fadR*Δ*mcrC*Δ*tyrR*Δ*ptsG*Δ*crr*Δ*pheA*, pETDuet-T7-*FjTAL*-*HpaBC*-T7-*AtCOMT* and pACYCDuet-T7-*aroG^fbr^*-*tyrC*-T7-*4CL*-*DCS*-(GGGGS)_3_-*CURS1* | This study |
| Cur67 | *E. coli* BL21(DE3) Δ*pykF*Δ*ushA::*T7*-ribF-TcTAL*Δ*curA::srpB*Δ*fadR*Δ*mcrC*Δ*tyrR*Δ*ptsG*Δ*crr*Δ*pheA*, pETDuet-T7-*FjTAL*-*HpaBC*-T7-*AtCOMT* and pCDFuet-T7-*aroG^fbr^*-*tyrC*-T7-*4CL*-*DCS*-(GGGGS)_3_-*CURS1* | This study |
| Cur68 | *E. coli* BL21(DE3) Δ*pykF*Δ*ushA::*T7*-ribF-TcTAL*Δ*curA::srpB*Δ*fadR*Δ*mcrC*Δ*tyrR*Δ*ptsG*Δ*crr*Δ*pheA*, pETDuet-T7-*FjTAL*-*HpaBC*-T7-*AtCOMT* and pRSFDuet-T7-*aroG^fbr^*-*tyrC*-T7-*4CL*-*DCS*-(GGGGS)_3_-*CURS1* | This study |
| Cur69 | *E. coli* BL21(DE3) Δ*pykF*Δ*ushA::*T7*-ribF-TcTAL*Δ*curA::srpB*Δ*fadR*Δ*mcrC*Δ*tyrR*Δ*ptsG*Δ*crr*Δ*pheA*, pRSFDuet-T7-*FjTAL*-*HpaBC*-T7-*AtCOMT* and pACYCDuet-T7-*aroG^fbr^*-*tyrC*-T7-*4CL*-*DCS*-(GGGGS)_3_-*CURS1* | This study |
| Cur70 | *E. coli* BL21(DE3) Δ*pykF*Δ*ushA::*T7*-ribF-TcTAL*Δ*curA::srpB*Δ*fadR*Δ*mcrC*Δ*tyrR*Δ*ptsG*Δ*crr*Δ*pheA*, pRSFDuet-T7-*FjTAL*-*HpaBC*-T7-*AtCOMT* and pCDFuet-T7-*aroG^fbr^*-*tyrC*-T7-*4CL*-*DCS*-(GGGGS)_3_-*CURS1* | This study |
| Cur71 | *E. coli* BL21(DE3) Δ*pykF*Δ*ushA::*T7*-ribF-TcTAL*Δ*curA::srpB*Δ*fadR*Δ*mcrC*Δ*tyrR*Δ*ptsG*Δ*crr*Δ*pheA*, pRSFDuet-T7-*FjTAL*-*HpaBC*-T7-*AtCOMT* and pETDuet-T7-*aroG^fbr^*-*tyrC*-T7-*4CL*-*DCS*-(GGGGS)_3_-*CURS1* | This study |
| Cur72 | *E. coli* BL21(DE3)-M1 Δ*curA::srpB*Δ*fadR*Δ*mcrC*Δ*tyrR*Δ*ptsG*Δ*crr*Δ*pheA*, pCDFDuet-T7-*ribF*-T7-*TcTAL* pACYCDuet-T7-*FjTAL*-*EcHpaBC*-T7-*AtCOMT* and pETDuet-T7-*aroG^fbr^*-*tyrC*-T7-*4CL*-*DCS*-(GGGGS)_3_-*CURS1* | This study |
| Cur73 | *E. coli* BL21(DE3)-M4 Δ*curA::srpB*Δ*fadR*Δ*mcrC*Δ*tyrR*Δ*ptsG*Δ*crr*Δ*pheA*, pCDFDuet-T7-*ribF*-T7-*TcTAL* pACYCDuet-T7-*FjTAL*-*EcHpaBC*-T7-*AtCOMT* and pETDuet-T7-*aroG^fbr^*-*tyrC*-T7-*4CL*-*DCS*-(GGGGS)_3_-*CURS1* | This study |
| Cur74 | *E. coli* BL21(DE3)-M14 Δ*curA::srpB*Δ*fadR*Δ*mcrC*Δ*tyrR*Δ*ptsG*Δ*crr*Δ*pheA*, pCDFDuet-T7-*ribF*-T7-*TcTAL* pACYCDuet-T7-*FjTAL*-*EcHpaBC*-T7-*AtCOMT* and pETDuet-T7-*aroG^fbr^*-*tyrC*-T7-*4CL*-*DCS*-(GGGGS)_3_-*CURS1* | This study |
| Cur75 | *E. coli* BL21(DE3)-M15 Δ*curA::srpB*Δ*fadR*Δ*mcrC*Δ*tyrR*Δ*ptsG*Δ*crr*Δ*pheA*, pCDFDuet-T7-*ribF*-T7-*TcTAL* pACYCDuet-T7-*FjTAL*-*EcHpaBC*-T7-*AtCOMT* and pETDuet-T7-*aroG^fbr^*-*tyrC*-T7-*4CL*-*DCS*-(GGGGS)_3_-*CURS1* | This study |
| Cur76 | *E. coli* BL21(DE3)-M24 Δ*curA::srpB*Δ*fadR*Δ*mcrC*Δ*tyrR*Δ*ptsG*Δ*crr*Δ*pheA*, pCDFDuet-T7-*ribF*-T7-*TcTAL* pACYCDuet-T7-*FjTAL*-*EcHpaBC*-T7-*AtCOMT* and pETDuet-T7-*aroG^fbr^*-*tyrC*-T7-*4CL*-*DCS*-(GGGGS)_3_-*CURS1* | This study |
| Cur77 | *E. coli* BL21(DE3)-M25 Δ*curA::srpB*Δ*fadR*Δ*mcrC*Δ*tyrR*Δ*ptsG*Δ*crr*Δ*pheA*, pCDFDuet-T7-*ribF*-T7-*TcTAL* pACYCDuet-T7-*FjTAL*-*EcHpaBC*-T7-*AtCOMT* and pETDuet-T7-*aroG^fbr^*-*tyrC*-T7-*4CL*-*DCS*-(GGGGS)_3_-*CURS1* | This study |
| Cur78 | *E. coli* BL21(DE3)-M26 Δ*curA::srpB*Δ*fadR*Δ*mcrC*Δ*tyrR*Δ*ptsG*Δ*crr*Δ*pheA*, pCDFDuet-T7-*ribF*-T7-*TcTAL* pACYCDuet-T7-*FjTAL*-*EcHpaBC*-T7-*AtCOMT* and pETDuet-T7-*aroG^fbr^*-*tyrC*-T7-*4CL*-*DCS*-(GGGGS)_3_-*CURS1* | This study |
| Cur79 | *E. coli* BL21(DE3)-M28 Δ*curA::srpB*Δ*fadR*Δ*mcrC*Δ*tyrR*Δ*ptsG*Δ*crr*Δ*pheA*, pCDFDuet-T7-*ribF*-T7-*TcTAL* pACYCDuet-T7-*FjTAL*-*EcHpaBC*-T7-*AtCOMT* and pETDuet-T7-*aroG^fbr^*-*tyrC*-T7-*4CL*-*DCS*-(GGGGS)_3_-*CURS1* | This study |
| Cur80 | *E. coli* BL21(DE3)-M29 Δ*curA::srpB*Δ*fadR*Δ*mcrC*Δ*tyrR*Δ*ptsG*Δ*crr*Δ*pheA*, pCDFDuet-T7-*ribF*-T7-*TcTAL* pACYCDuet-T7-*FjTAL*-*EcHpaBC*-T7-*AtCOMT* and pETDuet-T7-*aroG^fbr^*-*tyrC*-T7-*4CL*-*DCS*-(GGGGS)_3_-*CURS1* | This study |
| Cur81 | *E. coli* BL21(DE3)-M31 Δ*curA::srpB*Δ*fadR*Δ*mcrC*Δ*tyrR*Δ*ptsG*Δ*crr*Δ*pheA*, pCDFDuet-T7-*ribF*-T7-*TcTAL* pACYCDuet-T7-*FjTAL*-*EcHpaBC*-T7-*AtCOMT* and pETDuet-T7-*aroG^fbr^*-*tyrC*-T7-*4CL*-*DCS*-(GGGGS)_3_-*CURS1* | This study |
| Cur82 | *E. coli* BL21(DE3)-M34 Δ*curA::srpB*Δ*fadR*Δ*mcrC*Δ*tyrR*Δ*ptsG*Δ*crr*Δ*pheA*, pCDFDuet-T7-*ribF*-T7-*TcTAL* pACYCDuet-T7-*FjTAL*-*EcHpaBC*-T7-*AtCOMT* and pETDuet-T7-*aroG^fbr^*-*tyrC*-T7-*4CL*-*DCS*-(GGGGS)_3_-*CURS1* | This study |
| Cur83 | *E. coli* BL21(DE3)-M40 Δ*curA::srpB*Δ*fadR*Δ*mcrC*Δ*tyrR*Δ*ptsG*Δ*crr*Δ*pheA*, pCDFDuet-T7-*ribF*-T7-*TcTAL* pACYCDuet-T7-*FjTAL*-*EcHpaBC*-T7-*AtCOMT* and pETDuet-T7-*aroG^fbr^*-*tyrC*-T7-*4CL*-*DCS*-(GGGGS)_3_-*CURS1* | This study |
| Cur84 | *E. coli* BL21(DE3)-M26 Δ*pykF*Δ*ushA::*T7*-ribF-TcTAL*Δ*curA::srpB*Δ*fadR*Δ*mcrC*Δ*tyrR*Δ*ptsG*Δ*crr*Δ*pheA*, pACYCDuet-T7-*FjTAL*-*EcHpaBC*-T7-*AtCOMT* and pETDuet-T7-*aroG^fbr^*-*tyrC*-T7-*4CL*-*DCS*-(GGGGS)_3_-*CURS1* | This study |
| Cur85 | *E. coli* BL21(DE3)-M26 Δ*pykF*Δ*ushA::*T7*-ribF-TcTAL*Δ*curA::srpB*Δ*fadR*Δ*mcrC*Δ*tyrR*Δ*ptsG*Δ*crr*Δ*pheA*, pACYCDuet-T7-*RgTAL*-*EcHpaBC*-T7-*AtCOMT* and pETDuet-T7-*aroG^fbr^*-*tyrC*-T7-*4CL*-*DCS*-(GGGGS)_3_-*CURS1* | This study |
| Cur86 | *E. coli* BL21(DE3)-M26 Δ*pykF*Δ*ushA::*T7*-ribF-TcTAL*Δ*curA::srpB*Δ*fadR*Δ*mcrC*Δ*tyrR*Δ*ptsG*Δ*crr*Δ*pheA*, pACYCDuet-T7-*TcTAL*-*EcHpaBC*-T7-*AtCOMT* and pETDuet-T7-*aroG^fbr^*-*tyrC*-T7-*4CL*-*DCS*-(GGGGS)_3_-*CURS1* | This study |
| Cur87 | *E. coli* BL21(DE3)-M26 Δ*pykF*Δ*ushA::*T7*-ribF-TcTAL*Δ*curA::srpB*Δ*fadR*Δ*mcrC*Δ*tyrR*Δ*ptsG*Δ*crr*Δ*pheA*, pACYCDuet-T7-*FjTAL*-*CipBEcHpaB-CipBEcHpaC*-T7-*AtCOMT* and pETDuet-T7-*aroG^fbr^*-*tyrC*-T7-*4CL*-*DCS*-(GGGGS)_3_-*CURS1* | This study |
| Cur88 | *E. coli* BL21(DE3)-M26 Δ*pykF*Δ*ushA::*T7*-ribF-TcTAL*Δ*curA::srpB*Δ*fadR*Δ*mcrC*Δ*tyrR*Δ*ptsG*Δ*crr*Δ*pheA*, pACYCDuet-T7-*CipBFjTAL*-*CipBEcHpaB-EcHpaC*-T7-*AtCOMT* and pETDuet-T7-*aroG^fbr^*-*tyrC*-T7-*4CL*-*DCS*-(GGGGS)_3_-*CURS1* | This study |
| Cur89 | *E. coli* BL21(DE3)-M26 Δ*pykF*Δ*ushA::*T7*-ribF-TcTAL*Δ*curA::srpB*Δ*fadR*Δ*mcrC*Δ*tyrR*Δ*ptsG*Δ*crr*Δ*pheA*, pACYCDuet-T7-*CipBFjTAL*-*CipBEcHpaB-CipBEcHpaC*-T7-*AtCOMT* and pETDuet-T7-*aroG^fbr^*-*tyrC*-T7-*4CL*-*DCS*-(GGGGS)_3_-*CURS1* | This study |
| Cur90 | *E. coli* BL21(DE3)-M26 Δ*pykF*Δ*ushA::*T7*-ribF-TcTAL*Δ*curA::srpB*Δ*fadR*Δ*mcrC*Δ*tyrR*Δ*ptsG*Δ*crr*Δ*pheA*, pACYCDuet-T7-*FjTAL*-*PpHpaBC*-T7-*AtCOMT* and pETDuet-T7-*aroG^fbr^*-*tyrC*-T7-*4CL*-*DCS*-(GGGGS)_3_-*CURS1* | This study |
| Cur91 | *E. coli* BL21(DE3) -M26 Δ*pykF*Δ*ushA::*T7*-ribF-TcTAL*Δ*curA::srpB*Δ*fadR*Δ*mcrC*Δ*tyrR*Δ*ptsG*Δ*crr*Δ*pheA*, pACYCDuet-T7-*FjTAL*-*KpHpaBC*-T7-*AtCOMT* and pETDuet-T7-*aroG^fbr^*-*tyrC*-T7-*4CL*-*DCS*-(GGGGS)_3_-*CURS1* | This study |
| Cur92 | *E. coli* BL21(DE3)-M26 Δ*pykF*Δ*ushA::*T7*-ribF-TcTAL*Δ*curA::srpB*Δ*fadR*Δ*mcrC*Δ*tyrR*Δ*ptsG*Δ*crr*Δ*pheA*, pACYCDuet-T7-*FjTAL*-*RoHpaBC*-T7-*AtCOMT* and pETDuet-T7-*aroG^fbr^*-*tyrC*-T7-*4CL*-*DCS*-(GGGGS)_3_-*CURS1* | This study |
| Cur93 | *E. coli* BL21(DE3)-M26 Δ*pykF*Δ*ushA::*T7*-ribF-TcTAL*Δ*curA::srpB*Δ*fadR*Δ*mcrC*Δ*tyrR*Δ*ptsG*Δ*crr*Δ*pheA*, pACYCDuet-T7-*FjTAL*-*PaHpaBC*-T7-*AtCOMT* and pETDuet-T7-*aroG^fbr^*-*tyrC*-T7-*4CL*-*DCS*-(GGGGS)_3_-*CURS1* | This study |
| Cur94 | *E. coli* BL21(DE3) -M26 Δ*pykF*Δ*ushA::*T7*-ribF-TcTAL*Δ*curA::srpB*Δ*fadR*Δ*mcrC*Δ*tyrR*Δ*ptsG*Δ*crr*Δ*pheA*, pACYCDuet-T7-*FjTAL*-*TtHpaBC*-T7-*AtCOMT* and pETDuet-T7-*aroG^fbr^*-*tyrC*-T7-*4CL*-*DCS*-(GGGGS)_3_-*CURS1* | This study |
| Cur95 | *E. coli* BL21(DE3) -M26 Δ*pykF*Δ*ushA::*T7*-ribF-TcTAL*Δ*curA::srpB*Δ*fadR*Δ*mcrC*Δ*tyrR*Δ*ptsG*Δ*crr*Δ*pheA*, pACYCDuet-T7-*FjTAL*-T7-*KpHpaBC*-T7-*AtCOMT* and pETDuet-T7-*aroG^fbr^*-*tyrC*-T7-*4CL*-*DCS*-(GGGGS)_3_-*CURS1* | This study |
| Cur96 | *E. coli* BL21(DE3) -M26 Δ*pykF*Δ*ushA::*T7*-ribF-TcTAL*Δ*curA::srpB*Δ*fadR*Δ*mcrC*Δ*tyrR*Δ*ptsG*Δ*crr*Δ*pheA*, pACYCDuet-T7-*FjTAL*-*SeC3H*-*PpHpaC*-T7-*AtCOMT* and pETDuet-T7-*aroG^fbr^*-*tyrC*-T7-*4CL*-*DCS*-(GGGGS)_3_-*CURS1* | This study |
| Cur97 | *E. coli* BL21(DE3) -M26 Δ*pykF*Δ*ushA::*T7*-ribF-TcTAL*Δ*curA::srpB*Δ*fadR*Δ*mcrC*Δ*tyrR*Δ*ptsG*Δ*crr*Δ*pheA*, pACYCDuet-T7-*FjTAL*-*SeC3H*-*PaHpaC*-T7-*AtCOMT* and pETDuet-T7-*aroG^fbr^*-*tyrC*-T7-*4CL*-*DCS*-(GGGGS)_3_-*CURS1* | This study |
| Cur98 | *E. coli* BL21(DE3) -M26 Δ*pykF*Δ*ushA::*T7*-ribF-TcTAL*Δ*curA::srpB*Δ*fadR*Δ*mcrC*Δ*tyrR*Δ*ptsG*Δ*crr*Δ*pheA*, pACYCDuet-T7-*FjTAL*-*EcHpaB-KpHpaC-*T7-*AtCOMT* and pETDuet-T7-*aroG^fbr^*-*tyrC*-T7-*4CL*-*DCS*-(GGGGS)_3_-*CURS1* | This study |
| Cur99 | *E. coli* BL21(DE3) -M26 Δ*pykF*Δ*ushA::*T7*-ribF-TcTAL*Δ*curA::srpB*Δ*fadR*Δ*mcrC*Δ*tyrR*Δ*ptsG*Δ*crr*Δ*pheA*, pACYCDuet-T7-*FjTAL*-*EcHpaB-PaHpaC-*T7-*AtCOMT* and pETDuet-T7-*aroG^fbr^*-*tyrC*-T7-*4CL*-*DCS*-(GGGGS)_3_-*CURS1* | This study |
| Cur100 | *E. coli* BL21(DE3) -M26 Δ*pykF*Δ*ushA::*T7*-ribF-TcTAL*Δ*curA::srpB*Δ*fadR*Δ*mcrC*Δ*tyrR*Δ*ptsG*Δ*crr*Δ*pheA*, pACYCDuet-T7-*FjTAL*-*KpHpaB-EcHpaC-*T7-*AtCOMT* and pETDuet-T7-*aroG^fbr^*-*tyrC*-T7-*4CL*-*DCS*-(GGGGS)_3_-*CURS1* | This study |
| Cur101 | *E. coli* BL21(DE3) -M26 Δ*pykF*Δ*ushA::*T7*-ribF-TcTAL*Δ*curA::srpB*Δ*fadR*Δ*mcrC*Δ*tyrR*Δ*ptsG*Δ*crr*Δ*pheA*, pACYCDuet-T7-*FjTAL*-*KpHpaB-PaHpaC-*T7-*AtCOMT* and pETDuet-T7-*aroG^fbr^*-*tyrC*-T7-*4CL*-*DCS*-(GGGGS)_3_-*CURS1* | This study |
| Cur102 | *E. coli* BL21(DE3) -M26 Δ*pykF*Δ*ushA::*T7*-ribF-TcTAL*Δ*curA::srpB*Δ*fadR*Δ*mcrC*Δ*tyrR*Δ*ptsG*Δ*crr*Δ*pheA*, pACYCDuet-T7-*FjTAL*-*PaHpaB-EcHpaC-*T7-*AtCOMT* and pETDuet-T7-*aroG^fbr^*-*tyrC*-T7-*4CL*-*DCS*-(GGGGS)_3_-*CURS1* | This study |
| Cur103 | *E. coli* BL21(DE3) -M26 Δ*pykF*Δ*ushA::*T7*-ribF-TcTAL*Δ*curA::srpB*Δ*fadR*Δ*mcrC*Δ*tyrR*Δ*ptsG*Δ*crr*Δ*pheA*, pACYCDuet-T7-*FjTAL*-*PaHpaB-KpHpaC-*T7-*AtCOMT* and pETDuet-T7-*aroG^fbr^*-*tyrC*-T7-*4CL*-*DCS*-(GGGGS)_3_-*CURS1* | This study |
| Cur104 | *E. coli* BL21(DE3) -M26 Δ*pykF*Δ*ushA::*T7*-ribF-TcTAL*Δ*curA::srpB*Δ*fadR*Δ*mcrC*Δ*tyrR*Δ*ptsG*Δ*crr*Δ*pheA*, pACYCDuet-T7-*FjTAL-KpHpaC-*T7-*AtCOMT-maeB* and pETDuet-T7-*aroG^fbr^*-*tyrC*-T7-*4CL*-*DCS*-(GGGGS)_3_-*CURS1* | This study |
| Cur105 | *E. coli* BL21(DE3) -M26 Δ*pykF*Δ*ushA::*T7*-ribF-TcTAL*Δ*curA::srpB*Δ*fadR*Δ*mcrC*Δ*tyrR*Δ*ptsG*Δ*crr*Δ*pheA*, pACYCDuet-T7-*FjTAL*-*KpHpaBC-*T7-*AtCOMT-sthA* and pETDuet-T7-*aroG^fbr^*-*tyrC*-T7-*4CL*-*DCS*-(GGGGS)_3_-*CURS1* | This study |
| Cur106 | *E. coli* BL21(DE3) -M26 Δ*pykF*Δ*ushA::*T7*-ribF-TcTAL*Δ*curA::srpB*Δ*fadR*Δ*mcrC*Δ*tyrR*Δ*ptsG*Δ*crr*Δ*pheA*, pACYCDuet-T7-*FjTAL*-*KpHpaBC-*T7-*AtCOMT-fre* and pETDuet-T7-*aroG^fbr^*-*tyrC*-T7-*4CL*-*DCS*-(GGGGS)_3_-*CURS1* | This study |
| Cur107 | *E. coli* BL21(DE3) -M26 Δ*pykF*Δ*ushA::*T7*-ribF-TcTAL*Δ*curA::srpB*Δ*fadR*Δ*mcrC*Δ*tyrR*Δ*ptsG*Δ*crr*Δ*pheA*, pACYCDuet-T7-*FjTAL*-*KpHpaBC-*T7-*AtCOMT-zwf* and pETDuet-T7-*aroG^fbr^*-*tyrC*-T7-*4CL*-*DCS*-(GGGGS)_3_-*CURS1* | This study |
| Cur108 | *E. coli* BL21(DE3) -M26 Δ*pykF*Δ*ushA::*T7*-ribF-TcTAL*Δ*curA::srpB*Δ*fadR*Δ*mcrC*Δ*tyrR*Δ*ptsG*Δ*crr*Δ*pheA*, pACYCDuet-T7-*FjTAL*-*KpHpaBC-*T7-*AtCOMT-gdhA* and pETDuet-T7-*aroG^fbr^*-*tyrC*-T7-*4CL*-*DCS*-(GGGGS)_3_-*CURS1* | This study |
| Cur109 | *E. coli* BL21(DE3) -M26 Δ*pykF*Δ*ushA::*T7*-ribF-TcTAL*Δ*curA::srpB*Δ*fadR*Δ*mcrC*Δ*tyrR*Δ*ptsG*Δ*crr*Δ*pheA*, pACYCDuet-T7-*FjTAL*-*KpHpaBC-*T7-*AtCOMT-gnd* and pETDuet-T7-*aroG^fbr^*-*tyrC*-T7-*4CL*-*DCS*-(GGGGS)_3_-*CURS1* | This study |
| Cur110 | *E. coli* BL21(DE3) -M26 Δ*ndh*Δ*pykF*Δ*ushA::*T7*-ribF-TcTAL*Δ*curA::srpB*Δ*fadR*Δ*mcrC*Δ*tyrR*Δ*ptsG*Δ*crr*Δ*pheA*, pACYCDuet-T7-*FjTAL*-*KpHpaBC-*T7-*AtCOMT* and pETDuet-T7-*aroG^fbr^*-*tyrC*-T7-*4CL*-*DCS*-(GGGGS)_3_-*CURS1* | This study |
| Cur111 | *E. coli* BL21(DE3) -M26 Δ*nuoE*Δ*pykF*Δ*ushA::*T7*-ribF-TcTAL*Δ*curA::srpB*Δ*fadR*Δ*mcrC*Δ*tyrR*Δ*ptsG*Δ*crr*Δ*pheA*, pACYCDuet-T7-*FjTAL*-*KpHpaBC-*T7-*AtCOMT* and pETDuet-T7-*aroG^fbr^*-*tyrC*-T7-*4CL*-*DCS*-(GGGGS)_3_-*CURS1* | This study |
| Cur112 | *E. coli* BL21(DE3) -M26 Δ*kefF*Δ*pykF*Δ*ushA::*T7*-ribF-TcTAL*Δ*curA::srpB*Δ*fadR*Δ*mcrC*Δ*tyrR*Δ*ptsG*Δ*crr*Δ*pheA*, pACYCDuet-T7-*FjTAL*-*KpHpaBC-*T7-*AtCOMT* and pETDuet-T7-*aroG^fbr^*-*tyrC*-T7-*4CL*-*DCS*-(GGGGS)_3_-*CURS1* | This study |
| Cur113 | *E. coli* BL21(DE3) -M26 Δ*wrbA*Δ*pykF*Δ*ushA::*T7*-ribF-TcTAL*Δ*curA::srpB*Δ*fadR*Δ*mcrC*Δ*tyrR*Δ*ptsG*Δ*crr*Δ*pheA*, pACYCDuet-T7-*FjTAL*-*KpHpaBC-*T7-*AtCOMT* and pETDuet-T7-*aroG^fbr^*-*tyrC*-T7-*4CL*-*DCS*-(GGGGS)_3_-*CURS1* | This study |
| Cur114 | *E. coli* BL21(DE3) -M26 Δ*yieF*Δ*pykF*Δ*ushA::*T7*-ribF-TcTAL*Δ*curA::srpB*Δ*fadR*Δ*mcrC*Δ*tyrR*Δ*ptsG*Δ*crr*Δ*pheA*, pACYCDuet-T7-*FjTAL*-*KpHpaBC-*T7-*AtCOMT* and pETDuet-T7-*aroG^fbr^*-*tyrC*-T7-*4CL*-*DCS*-(GGGGS)_3_-*CURS1* | This study |
| Cur115 | *E. coli* BL21(DE3) -M26 Δ*ygiN*Δ*pykF*Δ*ushA::*T7*-ribF-TcTAL*Δ*curA::srpB*Δ*fadR*Δ*mcrC*Δ*tyrR*Δ*ptsG*Δ*crr*Δ*pheA*, pACYCDuet-T7-*FjTAL*-*KpHpaBC-*T7-*AtCOMT* and pETDuet-T7-*aroG^fbr^*-*tyrC*-T7-*4CL*-*DCS*-(GGGGS)_3_-*CURS1* | This study |
| Cur116 | *E. coli* BL21(DE3) -M26 Δ*ndh*Δ*mqo*Δ*pykF*Δ*ushA::*T7*-ribF-TcTAL*Δ*curA::srpB*Δ*fadR*Δ*mcrC*Δ*tyrR*Δ*ptsG*Δ*crr*Δ*pheA*, pACYCDuet-T7-*FjTAL*-*KpHpaBC-*T7-*AtCOMT* and pETDuet-T7-*aroG^fbr^*-*tyrC*-T7-*4CL*-*DCS*-(GGGGS)_3_-*CURS1* | This study |
| Cur117 | *E. coli* BL21(DE3) -M26 Δ*ndh*Δ*dld*Δ*pykF*Δ*ushA::*T7*-ribF-TcTAL*Δ*curA::srpB*Δ*fadR*Δ*mcrC*Δ*tyrR*Δ*ptsG*Δ*crr*Δ*pheA*, pACYCDuet-T7-*FjTAL*-*KpHpaBC-*T7-*AtCOMT* and pETDuet-T7-*aroG^fbr^*-*tyrC*-T7-*4CL*-*DCS*-(GGGGS)_3_-*CURS1* | This study |
| Cur118 | *E. coli* BL21(DE3) -M26 Δ*ndh*Δ*lldD*Δ*pykF*Δ*ushA::*T7*-ribF-TcTAL*Δ*curA::srpB*Δ*fadR*Δ*mcrC*Δ*tyrR*Δ*ptsG*Δ*crr*Δ*pheA*, pACYCDuet-T7-*FjTAL*-*KpHpaBC-*T7-*AtCOMT* and pETDuet-T7-*aroG^fbr^*-*tyrC*-T7-*4CL*-*DCS*-(GGGGS)_3_-*CURS1* | This study |
| Cur119 | *E. coli* BL21(DE3) -M26 Δ*ndh*Δ*poxB*Δ*pykF*Δ*ushA::*T7*-ribF-TcTAL*Δ*curA::srpB*Δ*fadR*Δ*mcrC*Δ*tyrR*Δ*ptsG*Δ*crr*Δ*pheA*, pACYCDuet-T7-*FjTAL*-*KpHpaBC-*T7-*AtCOMT* and pETDuet-T7-*aroG^fbr^*-*tyrC*-T7-*4CL*-*DCS*-(GGGGS)_3_-*CURS1* | This study |
| Cur120 | *E. coli* BL21(DE3) -M26 Δ*ndh*Δ*sdhC*Δ*pykF*Δ*ushA::*T7*-ribF-TcTAL*Δ*curA::srpB*Δ*fadR*Δ*mcrC*Δ*tyrR*Δ*ptsG*Δ*crr*Δ*pheA*, pACYCDuet-T7-*FjTAL*-*KpHpaBC-*T7-*AtCOMT* and pETDuet-T7-*aroG^fbr^*-*tyrC*-T7-*4CL*-*DCS*-(GGGGS)_3_-*CURS1* | This study |
| Cur121 | *E. coli* BL21(DE3) -M26 Δ*ndh*Δ*fadE*Δ*pykF*Δ*ushA::*T7*-ribF-TcTAL*Δ*curA::srpB*Δ*fadR*Δ*mcrC*Δ*tyrR*Δ*ptsG*Δ*crr*Δ*pheA*, pACYCDuet-T7-*FjTAL*-*KpHpaBC-*T7-*AtCOMT* and pETDuet-T7-*aroG^fbr^*-*tyrC*-T7-*4CL*-*DCS*-(GGGGS)_3_-*CURS1* | This study |
| Cur122 | *E. coli* BL21(DE3) -M26 Δ*yhhY*::Ptrc-VHBΔ*ndh*Δ*lldD*Δ*pykF*Δ*ushA::*T7*-ribF-TcTAL*Δ*curA::srpB*Δ*fadR*Δ*mcrC*Δ*tyrR*Δ*ptsG*Δ*crr*Δ*pheA*, pACYCDuet-T7-*FjTAL*-*KpHpaBC-*T7-*AtCOMT* and pETDuet-T7-*aroG^fbr^*-*tyrC*-T7-*4CL*-*DCS*-(GGGGS)_3_-*CURS1* | This study |
| Cur123 | *E. coli* BL21(DE3) -M26 Δ*yhhY*::PJ23119-VHBΔ*ndh*Δ*lldD*Δ*pykF*Δ*ushA::*T7*-ribF-TcTAL*Δ*curA::srpB*Δ*fadR*Δ*mcrC*Δ*tyrR*Δ*ptsG*Δ*crr*Δ*pheA*, pACYCDuet-T7-*FjTAL*-*KpHpaBC-*T7-*AtCOMT* and pETDuet-T7-*aroG^fbr^*-*tyrC*-T7-*4CL*-*DCS*-(GGGGS)_3_-*CURS1* | This study |
| Cur124 | *E. coli* BL21(DE3) -M26 Δ*yddG*::Ptrc-*aroP*Δ*yhhY*::PJ23119-VHBΔ*ndh*Δ*lldD*Δ*pykF*Δ*ushA::*T7*-ribF-TcTAL*Δ*curA::srpB*Δ*fadR*Δ*mcrC*Δ*tyrR*Δ*ptsG*Δ*crr*Δ*pheA*, pACYCDuet-T7-*FjTAL*-*KpHpaBC-*T7-*AtCOMT* and pETDuet-T7-*aroG^fbr^*-*tyrC*-T7-*4CL*-*DCS*-(GGGGS)_3_-*CURS1* | This study |
| Cur125 | *E. coli* BL21(DE3) -M26Δ*yddG*::PJ23119-*aroP*Δ*yhhY*::PJ23119-VHBΔ*ndh*Δ*lldD*Δ*pykF*Δ*ushA::*T7*-ribF-TcTAL*Δ*curA::srpB*Δ*fadR*Δ*mcrC*Δ*tyrR*Δ*ptsG*Δ*crr*Δ*pheA*, pACYCDuet-T7-*FjTAL*-*KpHpaBC-*T7-*AtCOMT* and pETDuet-T7-*aroG^fbr^*-*tyrC*-T7-*4CL*-*DCS*-(GGGGS)_3_-*CURS1* | This study |
| Cur126 | *E. coli* BL21(DE3) -M26 Δ*yddG*::Ptrc-*tyrP*Δ*yhhY*::PJ23119-VHBΔ*ndh*Δ*lldD*Δ*pykF*Δ*ushA::*T7*-ribF-TcTAL*Δ*curA::srpB*Δ*fadR*Δ*mcrC*Δ*tyrR*Δ*ptsG*Δ*crr*Δ*pheA*, pACYCDuet-T7-*FjTAL*-*KpHpaBC-*T7-*AtCOMT* and pETDuet-T7-*aroG^fbr^*-*tyrC*-T7-*4CL*-*DCS*-(GGGGS)_3_-*CURS1* | This study |
| Cur127 | *E. coli* BL21(DE3) -M26 Δ*yddG*::Ptrc-*tyrP*Δ*yhhY*::PJ23119-VHBΔ*ndh*Δ*lldD*Δ*pykF*Δ*ushA::*T7*-ribF-TcTAL*Δ*curA::srpB*Δ*fadR*Δ*mcrC*Δ*tyrR*Δ*ptsG*Δ*crr*Δ*pheA*, pACYCDuet-T7-*FjTAL*-*KpHpaBC-*T7-*AtCOMT* and pETDuet-T7-*aroG^fbr^*-*tyrC*-T7-*4CL*-*DCS*-(GGGGS)_3_-*CURS1* | This study |
| Cur128 | *E. coli* BL21(DE3) -M26 Δ*yddG*::Ptrc-*tyrP*Δ*yhhY*::PJ23119-VHBΔ*ndh*Δ*lldD*Δ*pykF*Δ*ushA::*T7*-ribF-TcTAL*Δ*curA::srpB*Δ*fadR*Δ*mcrC*Δ*tyrR*Δ*ptsG*Δ*crr*Δ*pheA*, pACYCDuet-T7-*FjTAL^V471A^*-*KpHpaBC-*T7-*AtCOMT* and pETDuet-T7-*aroG^fbr^*-*tyrC*-T7-*4CL*-*DCS*-(GGGGS)_3_-*CURS1* | This study |
| Cur129 | *E. coli* BL21(DE3) -M26 Δ*yddG*::Ptrc-*tyrP*Δ*yhhY*::PJ23119-VHBΔ*ndh*Δ*lldD*Δ*pykF*Δ*ushA::*T7*-ribF-TcTAL*Δ*curA::srpB*Δ*fadR*Δ*mcrC*Δ*tyrR*Δ*ptsG*Δ*crr*Δ*pheA*, pACYCDuet-T7-*FjTAL^K350R^*-*KpHpaBC-*T7-*AtCOMT* and pETDuet-T7-*aroG^fbr^*-*tyrC*-T7-*4CL*-*DCS*-(GGGGS)_3_-*CURS1* | This study |
| Cur130 | *E. coli* BL21(DE3) -M26 Δ*yddG*::Ptrc-*tyrP*Δ*yhhY*::PJ23119-VHBΔ*ndh*Δ*lldD*Δ*pykF*Δ*ushA::*T7*-ribF-TcTAL*Δ*curA::srpB*Δ*fadR*Δ*mcrC*Δ*tyrR*Δ*ptsG*Δ*crr*Δ*pheA*, pACYCDuet-T7-*FjTAL^V456(GTG-GTA)^*-*KpHpaBC-*T7-*AtCOMT* and pETDuet-T7-*aroG^fbr^*-*tyrC*-T7-*4CL*-*DCS*-(GGGGS)_3_-*CURS1* | This study |
| Cur131 | *E. coli* BL21(DE3) -M26 Δ*yddG*::Ptrc-*tyrP*Δ*yhhY*::PJ23119-VHBΔ*ndh*Δ*lldD*Δ*pykF*Δ*ushA::*T7*-ribF-TcTAL*Δ*curA::srpB*Δ*fadR*Δ*mcrC*Δ*tyrR*Δ*ptsG*Δ*crr*Δ*pheA*, pACYCDuet-T7-*FjTAL^L101S^*-*KpHpaBC-*T7-*AtCOMT* and pETDuet-T7-*aroG^fbr^*-*tyrC*-T7-*4CL*-*DCS*-(GGGGS)_3_-*CURS1* | This study |
| Cur132 | *E. coli* BL21(DE3) -M26 Δ*yddG*::Ptrc-*tyrP*Δ*yhhY*::PJ23119-VHBΔ*ndh*Δ*lldD*Δ*pykF*Δ*ushA::*T7*-ribF-TcTAL*Δ*curA::srpB*Δ*fadR*Δ*mcrC*Δ*tyrR*Δ*ptsG*Δ*crr*Δ*pheA*, pACYCDuet-T7-*FjTAL*-*KpHpaB^Q465H^C-*T7-*AtCOMT* and pETDuet-T7-*aroG^fbr^*-*tyrC*-T7-*4CL*-*DCS*-(GGGGS)_3_-*CURS1* | This study |
| Cur133 | *E. coli* BL21(DE3) -M26 Δ*yddG*::Ptrc-*tyrP*Δ*yhhY*::PJ23119-VHBΔ*ndh*Δ*lldD*Δ*pykF*Δ*ushA::*T7*-ribF-TcTAL*Δ*curA::srpB*Δ*fadR*Δ*mcrC*Δ*tyrR*Δ*ptsG*Δ*crr*Δ*pheA*, pACYCDuet-T7-*FjTAL*-*KpHpaB^F450Y/K440N^C-*T7-*AtCOMT* and pETDuet-T7-*aroG^fbr^*-*tyrC*-T7-*4CL*-*DCS*-(GGGGS)_3_-*CURS1* | This study |
| Cur134 | *E. coli* BL21(DE3) -M26 Δ*yddG*::Ptrc-*tyrP*Δ*yhhY*::PJ23119-VHBΔ*ndh*Δ*lldD*Δ*pykF*Δ*ushA::*T7*-ribF-TcTAL*Δ*curA::srpB*Δ*fadR*Δ*mcrC*Δ*tyrR*Δ*ptsG*Δ*crr*Δ*pheA*, pACYCDuet-T7-*FjTAL*-*KpHpaB^P3L/N141T/S358T^C-*T7-*AtCOMT* and pETDuet-T7-*aroG^fbr^*-*tyrC*-T7-*4CL*-*DCS*-(GGGGS)_3_-*CURS1* | This study |
| Cur135 | *E. coli* BL21(DE3) -M26 Δ*yddG*::Ptrc-*tyrP*Δ*yhhY*::PJ23119-VHBΔ*ndh*Δ*lldD*Δ*pykF*Δ*ushA::*T7*-ribF-TcTAL*Δ*curA::srpB*Δ*fadR*Δ*mcrC*Δ*tyrR*Δ*ptsG*Δ*crr*Δ*pheA*, pACYCDuet-T7-*FjTAL*-*KpHpaB^M214K^C-*T7-*AtCOMT* and pETDuet-T7-*aroG^fbr^*-*tyrC*-T7-*4CL*-*DCS*-(GGGGS)_3_-*CURS1* | This study |
| Cur136 | *E. coli* BL21(DE3) -M26 Δ*yddG*::Ptrc-*tyrP*Δ*yhhY*::PJ23119-VHBΔ*ndh*Δ*lldD*Δ*pykF*Δ*ushA::*T7*-ribF-TcTAL*Δ*curA::srpB*Δ*fadR*Δ*mcrC*Δ*tyrR*Δ*ptsG*Δ*crr*Δ*pheA*, pACYCDuet-T7-*FjTAL*-*KpHpaB^Q465H/F450Y/K440N^C*  *-*T7-*AtCOMT* and pETDuet-T7-*aroG^fbr^*-*tyrC*-T7-*4CL*-*DCS*-(GGGGS)_3_-*CURS1* | This study |
| Cur137 | *E. coli* BL21(DE3) -M26 Δ*yddG*::Ptrc-*tyrP*Δ*yhhY*::PJ23119-VHBΔ*ndh*Δ*lldD*Δ*pykF*Δ*ushA::*T7*-ribF-TcTAL*Δ*curA::srpB*Δ*fadR*Δ*mcrC*Δ*tyrR*Δ*ptsG*Δ*crr*Δ*pheA*, pACYCDuet-T7-*FjTAL^L101S^*  -*KpHpaB ^Q465H^C-*T7-*AtCOMT* and pETDuet-T7-*aroG^fbr^*-*tyrC*-T7-*4CL*-*DCS*-(GGGGS)_3_-*CURS1* | This study |
| Cur138 | *E. coli* BL21(DE3) -M26 Δ*yddG*::Ptrc-*tyrP*Δ*yhhY*::PJ23119-VHBΔ*ndh*Δ*lldD*Δ*pykF*Δ*ushA::*T7*-ribF-TcTAL*Δ*curA::srpB*Δ*fadR*Δ*mcrC*Δ*tyrR*Δ*ptsG*Δ*crr*Δ*pheA*, pACYCDuet-T7-*FjTAL^L101S^*  -*KpHpaB^F450Y/K440N^C-*T7-*AtCOMT* and pETDuet-T7-*aroG^fbr^*-*tyrC*-T7-*4CL*-*DCS*-(GGGGS)_3_-*CURS1* | This study |
| CA24 | *E. coli* BL21 (DE3) Δ*pykF*Δ*ushA::*T7*-ribF-TcTAL*Δ*curA::srpB*Δ*fadR*Δ*mcrC*Δ*tyrR*Δ*crr*Δ*ptsG*Δ*pheA* containing pETDuet-*aroG*^fbr^-*tyrC* and pACYCDuet-*FjTAL*-RBS-*EcHpaBC*-*ycjP* | This study |
| CA25 | *E. coli* BL21(DE3)-M26 Δ*pykF*Δ*ushA::*T7*-ribF-TcTAL*Δ*curA::srpB*Δ*fadR*Δ*mcrC*Δ*tyrR*Δ*ptsG*Δ*crr*Δ*pheA*, pETDuet-*aroG*^fbr^-*tyrC* and pACYCDuet-*FjTAL*-RBS-*EcHpaBC*-*ycjP* | This study |
| CA26 | *E. coli* BL21(DE3)-M26 Δ*pykF*Δ*ushA::*T7*-ribF-TcTAL*Δ*curA::srpB*Δ*fadR*Δ*mcrC*Δ*tyrR*Δ*ptsG*Δ*crr*Δ*pheA*, pETDuet-*aroG*^fbr^-*tyrC* and pACYCDuet-*FjTAL*-RBS-*KpHpaBC*-*ycjP* | This study |
| CA27 | *E. coli* BL21(DE3) -M26 Δ*ndh*Δ*dld*Δ*pykF*Δ*ushA::*T7*-ribF-TcTAL*Δ*curA::srpB*Δ*fadR*Δ*mcrC*Δ*tyrR*Δ*ptsG*Δ*crr*Δ*pheA*, pETDuet-*aroG*^fbr^-*tyrC* and pACYCDuet-*FjTAL*-RBS-*KpHpaBC*-*ycjP* | This study |
| CA28 | *E. coli* BL21(DE3) -M26 Δ*ndh*Δ*dld*Δ*pykF*Δ*ushA::*T7*-ribF-TcTAL*Δ*curA::srpB*Δ*fadR*Δ*mcrC*Δ*tyrR*Δ*ptsG*Δ*crr*Δ*pheA*, pETDuet-*aroG*^fbr^-*tyrC* and pACYCDuet-*FjTAL*-RBS-*KpHpaB^Q465H^C*-*ycjP* | This study |
| CA29 | *E. coli* BL21(DE3) -M26 Δ*yhhY*::PJ23119-VHBΔ*ndh*Δ*lldD*Δ*pykF*Δ*ushA::*T7*-ribF-TcTAL*Δ*curA::srpB*Δ*fadR*Δ*mcrC*Δ*tyrR*Δ*ptsG*Δ*crr*Δ*pheA*, pETDuet-*aroG*^fbr^-*tyrC* and pACYCDuet-*FjTAL*-RBS-*KpHpaB^Q465H^C*-*ycjP* | This study |
| CA30 | *E. coli* BL21(DE3) -M26 Δ*yddG*::Ptrc-*tyrP*Δ*yhhY*::PJ23119-VHBΔ*ndh*Δ*lldD*Δ*pykF*Δ*ushA::*T7*-ribF-TcTAL*Δ*curA::srpB*Δ*fadR*Δ*mcrC*Δ*tyrR*Δ*ptsG*Δ*crr*Δ*pheA*, pETDuet-*aroG*^fbr^-*tyrC* and pACYCDuet-*FjTAL*-RBS-*KpHpaB^Q465H^C*-*ycjP* | This study |

## Table S2. Primers used in this study.

| **Primer** | **Sequence (5'-3')** |  |
| --- | --- | --- |
| ushA-UP-F | GCTTAAGGTAAAGCACATGCCAATTGG |  |
| ushA-UP-R | CATTATGCGGCCGCAAGCTTACTTCTCTCCCTGACCTGATTTCAAC |  |
| T7-TcTAL-ribF-F | AAGCTTGCGGCCGCATAATG |  |
| T7-TcTAL-ribF-R | GCACTTTCGGACAAAAAACCCCTCAAGACCCGTT |  |
| ushA-DOWN-F | GCATAACCCCTTGGGGCCTCTAAACGGGTCTTGAGGGGTTTTTTGTCCGAAAGTGCCGGATGTTTG |  |
| ushA-DOWN-R | CCATGACACCCGCAGTTAAATTACTC |  |
| ushA-sgRNA20nt1-F | GGTGGATGGTATCCGCAAAGGTTTTAGAGCTAGAAATAGCAAGTT |  |
| ushA-sgRNA20nt1-R | CTTTGCGGATACCATCCACCACTAGTATTATACCTAGGACTGAGC |  |
| pheA -UP-F | GCTTAAGGTAAAGCACATGCCAATTGG |  |
| pheA -UP-R | CATTATGCGGCCGCAAGCTTACTTCTCTCCCTGACCTGATTTCAAC |  |
| pheA -sgRNA20nt1-F | GACATCGGAAAACCCGTTACGTTTTAGAGCTAGAAATAGCAAGTT |  |
| pheA -sgRNA20nt1-R | GTAACGGGTTTTCCGATGTCACTAGTATTATACCTAGGACTGAGC |  |
| pheA-DOWN-F | GAAAAGGTGCCGGATGATGTGAATCATCCG |  |
| pheA-DOWN-R | ACCCGATGTTCGGCCCGGACAGCG |  |
| pykF-UP-F | ATCAGCATATATCAATCTAACCAATTAAACAAAAATCAAA |  |
| pykF-UP-R | GACAGTCTTAGTCTTTAAGTTGAGAAGGATGGGAGAAA |  |
| pykF-sgRNA20nt1-F | GAGCACCTGAAAGCGCACGGGTTTTAGAGCTAGAAATAGCAAGTT |  |
| pykF-sgRNA20nt1-R | CCGTGCGCTTTCAGGTGCTCACTAGTATTATACCTAGGACTGAGC |  |
| pykF-DOWN-F | TGAATTAATTTGTATATCGAAGCGCCCTGATGGGCGCTTTT |  |
| pykF-DOWN-R | ACAGGTACTATTACTTGCGG |  |
| T7-ppsA-F | AAGCTTGCGGCCGCATAATG |  |
| T7-ppsA- R | GCACTTTCGGACAAAAAACCCCTCAAGACCCGTT | |
| yhhY-UP-F | ATCGCTGGTGAAATGGATATGGGA | |
| yhhY -UP-R | TCCTTTGCCCTCTTTGGGGTAAATGTCCCT | |
| yhhY -sgRNA20nt1-F | CAAAATCGGCAACATGACTGGTTTTAGAGCTAGAAATAGCAAGTT | |
| yhhY -sgRNA20nt1-R | CAGTCATGTTGCCGATTTTGACTAGTATTATACCTAGGACTGAGC | |
| yhhY -DOWN-F | AAGTTTCAGGCCGCTTTCAGGACATAAAAAACG | |
| yhhY -DOWN-R | GCCCCGTATTATTAATATTATTTAAGAGTGAGAATGTAAATG | |
| T7-vhb-F | AAGCTTGCGGCCGCATAATG | |
| T7-vhb-R | GCACTTTCGGACAAAAAACCCCTCAAGACCCGTT | |
| PT1-tyr-R | CCACCGCACCGGTCAGGACGTGTACTAGTATTATACCTAGGACTGAGCTAGCTGT | |
| PT1-tyr-F | ACACGTCCTGACCGGTGCGGTGGGTTTTAGAGCTAGAAATAGCAAGTTAAAATAAGGCTA | |
| ptsG-sgRNA20nt1-F | TCGTGATACGTTGGCTTCCAACTAGTATTATACCTAGGACTGAGC | |
| ptsG -sgRNA20nt1-R | TGGAAGCCAACGTATCACGAGTTTTAGAGCTAGAAATAGCAAGTT | |
| pykF-sgRNA20nt1-F | TCGCGGCATCGTAATCATATACTAGTATTATACCTAGGACTGAGC | |
| pykF-sgRNA20nt1-R | ATATGATTACGATGCCGCGAGTTTTAGAGCTAGAAATAGCAAGTT | |
| a26-pTarget-R-1 | CCGTGCGCTTTCAGGTGCTCACTAGTATTATACCTAGGACTGAGC | |
| a26-pTarget-F-1 | GAGCACCTGAAAGCGCACGGGTTTTAGAGCTAGAAATAGCAAGTT | |
| ptsG-up-F | GTTATGTCCCCCTGGATCGGTTACT | |
| ptsG-up-R | GTCGTATTAAATTGAGAGTGCTCCTGAGTATGGGTG | |
| T7-glk-F | GCACTCTCAATTTAATACGACTCACTATAGGGGA | |
| T7-glk-R | TCTGGCTGCCTTAGTCTCCCCAACGTCTTACAGACAAAAAAC | |
| ptsG-DOWN-F | GTAAGACGTTGGGGAGACTAAGGCAGCCAGATGGCTGCCTTTT | |
| ptsG-DOWN-R | CTATAAAGCGGTGGATGGGACA | |
| PTarget-∆yddG-1-R | AAAGGCTATTTACTCGCCGGACTAGTATTATACCTAGGACTGAGCTAGCTGT | |
| PTarget-∆yddG-1-F | CCGGCGAGTAAATAGCCTTTGTTTTAGAGCTAGAAATAGCAAGTTAAAATAAGGCTAGT | |
| pTarget-∆crr-1-R | GAGTCAGGGTAGACTTGGCTACTAGTATTATACCTAGGACTGAGC | |
| pTarget-∆crr-1-F | AGCCAAGTCTACCCTGACTCGTTTTAGAGCTAGAAATAGCAAGTT | |
| arop-UP-F | GCCTTTAAAATCATATTCCCAGTGACCAAAAC | |
| pJ-tyrp-UP-R | CTCCTCTTTGAATTCGCTAGCATTATACCTAGGACTGAGCTAGCTGTCAAAATAATTGCCGGGTCTACGG | |
| pJ-arop-F | CTAGGTATAATGCTAGCGAATTCAAAGAGGAGAAAGGATCCATGATGGAAGGTCAACAGCACGG | |
| pt-arop-R | GGCCCCAAGGGGTTATGCTAGTTATTGCTCAGCGGTGGCAGCAGCCTAGGTTAATTAATGCGCTTTTACGGCTTTGGC | |
| pt-tyrp-down-F | GCATAACCCCTTGGGGCCTCTAAACGGGTCTTGAGGGGTTTTTTGCGTGCTCCACAGGACGATCG | |
| pt-tyrp-down-R | CCATCACCCAGGCTATACATCAATAGCC | |
| ptrc-tyrp-UP-R | GCTCACAATTCCACACATTATACGAGCCGGATGATTAATTGTCAAAATAATTGCCGGGTCTACGG | |
| ptrc-arop-F | CGTATAATGTGTGGAATTGTGAGCGGATAACAATTTCACACAGGAAACAGACCATGATGGAAGGTCAACAGCACGG | |
| ptrc-tyrp-UP-F | GCCTTTAAAATCATATTCCCAGTGACCAAAAC | |
| pJ-tyrp-UP-R | CTCCTCTTTGAATTCGCTAGCATTATACCTAGGACTGAGCTAGCTGTCAAAATAATTGCCGGGTCTACGGC | |
| pJ-tyrp-F | CTAGGTATAATGCTAGCGAATTCAAAGAGGAGAAAGGATCCGTGAAAAACAGAACTCTGGGAAGTGTTTTT | |
|  |  | |
| pt-tyrP-R | GGCCCCAAGGGGTTATGCTAGTTATTGCTCAGCGGTGGCAGCAGCCTAGGTTAATCACCCCACTTCTGGTAACAACCC | |
| pt-tyrp-down-F | GCATAACCCCTTGGGGCCTCTAAACGGGTCTTGAGGGGTTTTTTGCGTGCTCCACAGGACGATCG | |
| pt-tyrp-down-R | CCATCACCCAGGCTATACATCAATAGCC | |
| ptrc-tyrp-F | CGTATAATGTGTGGAATTGTGAGCGGATAACAATTTCACACAGGAAACAGACCGTGAAAAACAGAACTCTGGGAAGTGTTTT | |

## Table S3. Acid resistant mutant26 target.

| **gene** | **mRNA ID** | **mRNA log2FC** | ***P*** | **protein log2FC** | ***P*** | **grade** | **mechanism** |
| --- | --- | --- | --- | --- | --- | --- | --- |
| Oats | HO397_RS18835 | 6.699117436 | 0.00459 | 1.541365 | 0.093907 | 5 | Organic acid transport system |
| rmuC | HO397_RS18855 | 6.31992784 | 2.21E-06 | 1.211944 | 0.03945 | 5 | DNA damage repair system |
| metE | HO397_RS18770 | 4.726243234 | 0.045615 | 0.283209 | 0.045077 | 4 | Amino acid metabolism system |
| glnA | HO397_RS19035 | 2.312149466 | 0.027412 | 0.339078 | 0.60443 | 4 | Glutamic acid acid resistant system |
| maoP | HO397_RS18425 | 1.580081367 | 0.014343 | 0.654294 | 0.067856 | 3 | Chromosome regulatory system |
| glnQ | HO397_RS03905 | 1.297588227 | 0.041215 | 0.303343 | 0.169249 | 3 | Glutamic acid acid resistant system |
| rbsB | HO397_RS18365 | 0.64078349 | 0.005489 | 0.986915 | 0.335347 | 2 | Carbon source transport system |

## Table S4. Differential expression of core metabolites related to acid tolerance

| **Metabolite name** | **KEGG ID** | **FC**  **(M15/ck)** | **FC (M26/ck)** | **M26 log2FC** | **M15**  **log2FC** | **M26 log2FC** | **pathway** |
| --- | --- | --- | --- | --- | --- | --- | --- |
| Palmitic Acid | - | 0.605 | 0.889 | -0.17 | -0.73 | -0.17 | fatty acid |
| 2-Amino-1,3,4-octadecanetriol | C12144 | 0.792 | 0.865 | -0.21 | -0.34 | -0.21 | fatty acid |
| L-Norleucine | C01933 | 1.145 | 1.456 | 0.54 | 0.2 | 0.54 | Amino acid metabolism system |
| Valine | C16436 | 1.214 | 1.355 | 0.44 | 0.28 | 0.44 | branched chain amino acid |
| Isoleucine | - | 1.226 | 0.88 | -0.18 | 0.29 | -0.18 | branched chain amino acid |
| DL-Arginine | C02385 | 1.408 | 1.807 | 0.85 | 0.49 | 0.85 | Arginine |
| L-Phenylalanine | C00079 | 9.515 | 10.466 | 3.39 | 3.25 | 3.39 | L-Phenylalanine |
| L-Lysine | C00047 | 2.765 | 3.584 | 1.84 | 1.47 | 1.84 | L-Lysine |
| DL-Tryptophan | C00806 | 3.072 | 3.933 | 1.98 | 1.62 | 1.98 | tryptophan |
| L-(-)-Methionine | C00073 | 1.538 | 1.983 | 0.99 | 0.62 | 0.99 | Methionine |
| D-(+)-Proline | C00763 | 3.367 | 3.903 | 1.96 | 1.75 | 1.96 | Proline |
| D-(+)-Pipecolinic acid | - | 2.937 | 4.132 | 2.05 | 1.55 | 2.05 | L-Glutamic acid |
| L-Pyroglutamic acid | C01879 | 1.414 | 1.649 | 0.72 | 0.5 | 0.72 | L-Glutamic acid |
| L-Glutamic acid | C00025 | 3.996 | 4.689 | 2.23 | 2 | 2.23 | L-Glutamic acid |
| L-Tyrosine | C00082 | 1.217 | 1.67 | 0.74 | 0.28 | 0.74 | L-Tyrosine |
| Nicotinamide | C00153 | 1.223 | 1.094 | 0.13 | 0.29 | 0.13 | nicotinic acid |
| Hypoxanthine | C00262 | 1.746 | 2.17 | 1.12 | 0.8 | 1.12 | purine |

## Table S5. Apparent kinetic parameters of *Kp*HpaB variants toward L-tyrosine and *p*-coumaric acid

| pH | Substrate | L-tyrosine | | | *p*-Coumaric acid | | |
| --- | --- | --- | --- | --- | --- | --- | --- |
|  | Enzyme | Km (uM) | Kcat (min^-1^) | Kcat/Km (uM^-1^·min^-1^) | Km (uM) | Kcat (min^-1^) | Kcat/Km (uM^-1^·min^-1^) |
| 5.7 | KpHpaB^WT^ | 194.73 | 703.83 | 3.61 | 291.23 | 434.09 | 1.49 |
|  | KpHpaB^Q465H^ | 487.64 | 293.85 | 0.60 | 131.32 | 851.54 | 6.48 |
| 6.0 | KpHpaB^WT^ | 128.83 | 764.98 | 5.94 | 304.01 | 579.94 | 1.91 |
|  | KpHpaB^Q465H^ | 446.10 | 336.03 | 0.75 | 118.30 | 1011.46 | 8.55 |
| 6.4 | KpHpaB^WT^ | 103.96 | 863.37 | 8.31 | 335.31 | 682.11 | 2.03 |
|  | KpHpaB^Q465H^ | 622.78 | 531.10 | 0.85 | 122.25 | 1071.72 | 8.77 |
| 7.5 | KpHpaB^WT^ | 98.35 | 939.70 | 9.55 | 244.60 | 687.80 | 2.81 |
|  | KpHpaB^Q465H^ | 555.1 | 620.00 | 1.12 | 136.90 | 956.00 | 6.98 |

## Table S6. Effect of ascorbic acid removal on curcumin production and A_400_-associated browning burden

| **Scale** | **Strain** | **Condition** | **Product** | **Titer (mg/L)** | **A_400_** | **FPI** | **rFPIᵢ** |
| --- | --- | --- | --- | --- | --- | --- | --- |
| Shake flask | Cur132 | + ascorbic acid | Curcumin | 233.6 | 1.13 | 206.73 | 1 |
| Shake flask | Cur132 | − ascorbic acid | Curcumin | 196.9 | 1.773 | 111.05 | 0.54 |
| 5 L bioreactor | Cur132 | pH–DO  + ascorbic acid | Curcumin | 1075.2 | 0.814 | 1320.88 | 1 |
| 5 L bioreactor | Cur132 | pH–DO  − ascorbic acid | Curcumin | 901.6 | 1.13 | 797.88 | 0.6 |

# Supplementary Figures

## Figure S1. Optimization of plasmid copy-number combinations for curcumin pathway expression.

Different replication-origin combinations were evaluated for the expression of curcumin biosynthetic modules. Curcumin titer, ferulic acid titer, and cell growth (OD_600_) were compared among the resulting strains. Data are presented as mean ± SD from three biological replicates.

## Figure S2. Spectral validation of A₄₀₀ as an operational indicator of L-DOPA-associated browning.

(A) UV–visible absorption spectra (300–500 nm) of saturated solutions of representative pathway-related compounds, including *p*-coumaric acid, caffeic acid, ferulic acid, curcumin, L-DOPA, L-tyrosine, and demethoxycurcumin, prepared in the fermentation-medium matrix; uninoculated medium was used as the control. (B) UV–visible spectra of L-DOPA-containing fermentation samples collected at different browning stages (0, 4, 6, 8, 10, and 22 h). Absorbance values exceeding the upper detection limit of the instrument were not plotted. (C) Time-dependent changes in residual L-DOPA concentration during incubation of culture medium supplemented with 5 mM L-DOPA, with medium lacking L-DOPA as the control. (D) Linear regression between L-DOPA consumption and browning intensity measured at 400 nm. The positive correlation indicates that L-DOPA consumption is associated with oxidation-related medium browning. Data in panels C and D are presented as mean ± SD from three independent biological replicates.

## Figure S3. Acidic conditions alleviate oxidative browning of medium containing 5 mM L-DOPA.

Browning of the medium at pH 7 and pH 4 was recorded at 0, 3, 6, 9, and 12 h. Under neutral conditions (pH 7), the medium exhibited progressive darkening and pronounced browning, whereas browning was markedly alleviated under acidic conditions (pH 4). These results indicate that L-3,4-dihydroxyphenylalanine (L-DOPA) is highly prone to oxidation and that low pH improves its stability.

## Figure S4. **Termination of lethality rate of ARTP-treated strains prior to MMC screening.**

(A) The lethality rate at different ARTP exposure times was determined by plate counting. Lethality increased with treatment duration and reached 97.6% at 60 s. Considering both mutation efficiency and survival rate, 60 s was selected as the optimal mutagenesis time for subsequent library construction and MMC screening. **(B)** Survival assessment of ARTP-treated cells under four serial dilution gradients (10^-1^ to 10^-4^), showing consistent lethality trends across dilution levels and further supporting the reliability of the selected treatment condition.

## Figure S5. Oxidative browning of L-DOPA is aggravated by increased substrate concentration and oxygen availability.

**(A–C)** Comparison of the browning behavior of 5 mM *p*-coumaric acid, 5 mM L-DOPA, and 5 mM caffeic acid at 0, 3, 6, 9, and 12 h. **(D, E)** Browning of 5 mM and 10 mM L-DOPA over the same time course, showing that higher initial L-DOPA concentration led to more severe oxidative browning. **(F)** Browning under different fermentation conditions, including CK (without L-DOPA addition), L (oxygen-limited shake-flask fermentation), N (normal shake-flask fermentation), and A (aerobic baffled-flask fermentation). Compared with oxygen-limited fermentation, browning was more pronounced under normal shake-flask conditions and was most severe under aerobic baffled-flask conditions, indicating that increased oxygen availability aggravated L-DOPA oxidation. These results demonstrate that L-DOPA is the most oxidation-prone intermediate among the tested compounds, supporting subsequent pathway optimization toward the *p*-coumaric acid branch and strain engineering for oxygen-limited fermentation.

## Figure S6. Effects of TAL variants from different sources on curcumin production in engineered strains.

## Figure S7. Introduction of CipB scaffold protein leads to conformational enzyme inactivation

## Figure S8. Respiratory-chain remodeling alters intracellular redox status and HpaBC-dependent hydroxylation capacity.

**(A)** Time-course changes in the intracellular NADH/(NADH + NAD⁺) ratio of Cur95 and the corresponding *ndh*/*lldD* deletion strains. **(B)** Time-course changes in intracellular ATP content. **(C)** Caffeic acid formation after 60 min in equal-biomass whole-cell hydroxylation assays using *p-*coumaric acid as the substrate. **(D)** Initial whole-cell hydroxylation rates normalized to dry cell weight (DCW). Data are presented as mean ± SD from three independent biological replicates.

## Figure S9. SDS-PAGE analysis of purified *Kp*HpaB variants and *Kp*HpaC.

Coomassie Brilliant Blue-stained SDS-PAGE gel showing the purified recombinant *Kp*HpaB^WT^, *Kp*HpaB^Q465H^, and *Kp*HpaC proteins used for in vitro enzymatic assays. The expected molecular weights of *Kp*HpaB^WT^ and *Kp*HpaB^Q465H^ are approximately 58.7 kDa, and that of *Kp*HpaC is approximately 18.4 kDa. The corresponding protein bands are indicated by red boxes.

## Figure S10. pH-dependent kinetic profiles of *Kp*HpaB^WT^ and *Kp*HpaB^Q465H^ toward *p*-coumaric acid and L-tyrosine. Michaelis–Menten kinetic analysis of *Kp*HpaB^WT^ and *Kp*HpaB^Q465H^ toward *p*-coumaric acid (pCA) and L-tyrosine (Tyr) at pH 5.7 (A), 6.0 (B), 6.4 (C), and 7.5 (D). At each pH, kinetic profiles are shown for WT and Q465H using pCA or Tyr as the substrate. The Q465H substitution consistently favored pCA hydroxylation while reducing activity toward Tyr across the tested pH range, supporting a pH-robust shift in substrate preference toward the productive branch. Data are presented as mean ± SD from three independent biological replicates.

## Figure S11. Effects of iterative combinatorial mutant construction on curcumin biosynthesis and the accumulation of pathway-related metabolites. Curcumin (Cur), ferulic acid (FA), caffeic acid (CA), *p*-coumaric acid (pCA), L-tyrosine (L-Tyr), and L-DOPA titers were measured in the wild type (WT) and engineered strains carrying individual or combined mutations in *Kp*HpaB and FjTAL. Cur, FA, CA, and pCA are shown on the left y-axis, whereas L-Tyr and L-DOPA are shown on the right y-axis. Data points represent independent biological replicates.

## Figure S12. Multiple sequence alignment of HpaB enzymes from different sources.

Amino acid sequences of HpaB homologs were aligned using Clustal Omega. Conserved residues are highlighted in blue. The consensus sequence is shown below each alignment block, and residue conservation at each position is represented by orange bar plots.TtHpaB from *Thermus thermophilus* (GenBank: AP025609.1); *Kp*HpaB, HpaB from *Klebsiella planticola* (Gene bank: CP180442.1). EcHpaB, HpaB from *Escherichia coli* (Gene bank: CP013031.1); PaHpaB, HpaB from *Pseudomonas aeruginosa* (Gene bank: GenBank: CP199216.1.); RoHpaB, HpaB from *Rhodococcus ruber* (GenBank: CP024315.1); PpHpaB, HpaB from *Pseudomonas putida* (GenBank: CP144123.1); SeC3H, HpaB from *Rhodococcus ruber* (GenBank: CP024315.1).

## Figure S13. High-throughput screening of FjTAL and *Kp*HpaB mutant libraries.

**(A)** Fermentation performance of the FjTAL mutant library, showing the relationship between ferulic acid and curcumin titers. **(B)** Distribution of curcumin titer normalized by OD_600_ in the FjTAL mutant library. **(C)** Fermentation performance of the *Kp*HpaB mutant library, illustrating the correlation between ferulic acid titer and curcumin titer. **(D)** Distribution of curcumin titer normalized by OD_600_ in the *Kp*HpaB mutant library. In all panels, the horizontal and vertical dashed lines indicate the wild-type reference.

## Figure S14. Gibbs free-energy landscapes of *Kp*HpaB^WT^ and *Kp*HpaB^Q465H^.

Gibbs free-energy landscapes projected onto the first two principal components (PC1 and PC2) for *Kp*HpaB^WT^ **(A)** and *Kp*HpaB^Q465H^ **(B)** during molecular dynamics simulations. The color scale represents the relative Gibbs free energy (kJ/mol), with lower-energy regions corresponding to more populated conformational states. The Q465H substitution altered the distribution of conformational energy basins, indicating remodeling of the conformational landscape relative to the WT enzyme.

## Figure S15. Fed-batch fermentation dynamics and A_400_-based oxidation burden under different process-control strategies.

**(A)** Fermentation performance of strain Cur84 under conventional control conditions. **(B)** Fermentation performance of strain Cur132 under the pH single-factor multistage control strategy. **(C)** Dynamic changes in oxidation burden during fed-batch fermentation under different strategies. **(D)** Time-course profiles of caffeic acid production, biomass accumulation, and A_400_ of CA30 under the original fermentation process. Results are mean ± SD from three independent experiments.

## Figure S16. Characterization of curcuminoid composition in shake-flask and 5 L bioreactor fermentation.

**(A)** HPLC retention profiles of authentic curcumin, demethoxycurcumin, and bisdemethoxycurcumin standards, together with extracts from shake-flask and 5 L bioreactor fermentations. **(B)** Relative composition of curcuminoids in the corresponding fermentation extracts. Curcumin accounted for 95.0% and 84.3% of total detected curcuminoids in shake-flask and 5 L bioreactor samples, respectively, whereas demethoxycurcumin accounted for 5.0% and 15.7%; bisdemethoxycurcumin was not detected. **(C)** LC–MS spectra of curcumin identified in the 5 L fermentation broth under positive- and negative-ion modes. **(D)** LC–MS spectra of demethoxycurcumin identified in the 5 L fermentation broth under positive- and negative-ion modes.

**References**

1.	Wang, L.; Li, N.; Yu, S.; Zhou, J., Enhancing caffeic acid production in *Escherichia coli* by engineering the biosynthesis pathway and transporter. *Bioresour Technol* **2023,** *368*, 128320.

2.	Chen, J.; Wang, W.; Wang, L.; Wang, H.; Hu, M.; Zhou, J.; Du, G.; Zeng, W., Efficient *De Novo* Biosynthesis of Curcumin in *Escherichia coli* by Optimizing Pathway Modules and Increasing the Malonyl-CoA Supply. *Journal of Agricultural and Food Chemistry* **2024,** *72* (1), 566-576.
